# Modelling renal defects in Bardet-Biedl syndrome patients using human iPS cells

**DOI:** 10.3389/fcell.2023.1163825

**Published:** 2023-06-02

**Authors:** James Williams, Chloe Hurling, Sabrina Munir, Peter Harley, Carolina Barcellos Machado, Ana-Maria Cujba, Mario Alvarez-Fallas, Davide Danovi, Ivo Lieberam, Rocio Sancho, Philip Beales, Fiona M. Watt

**Affiliations:** ^1^ Centre for Gene Therapy and Regenerative Medicine, King’s College London, Guy’s Hospital, London, United Kingdom; ^2^ Bit.bio, Babraham Research Campus, Cambridge, United Kingdom; ^3^ Centre for Developmental Neurobiology and MRC Centre for Neurodevelopmental Disorders, King’s College London, London, United Kingdom; ^4^ Institute of Child Health, Genetic and Genomic Medicine, University College London, London, United Kingdom

**Keywords:** iPSC (induced pluripotent stem cell), kidney, ciliopathy, CRISPR, Bardet-Biedl syndrome

## Abstract

Bardet-Biedl syndrome (BBS) is a ciliopathy with pleiotropic effects on multiple tissues, including the kidney. Here we have compared renal differentiation of iPS cells from healthy and BBS donors. High content image analysis of WT1-expressing kidney progenitors showed that cell proliferation, differentiation and cell shape were similar in healthy, *BBS1*, *BBS2*, and *BBS10* mutant lines. We then examined three patient lines with *BBS10* mutations in a 3D kidney organoid system. The line with the most deleterious mutation, with low BBS10 expression, expressed kidney marker genes but failed to generate 3D organoids. The other two patient lines expressed near normal levels of *BBS10* mRNA and generated multiple kidney lineages within organoids when examined at day 20 of organoid differentiation. However, on prolonged culture (day 27) the proximal tubule compartment degenerated. Introducing wild type *BBS10* into the most severely affected patient line restored organoid formation, whereas CRISPR-mediated generation of a truncating BBS10 mutation in a healthy line resulted in failure to generate organoids. Our findings provide a basis for further mechanistic studies of the role of BBS10 in the kidney.

## Introduction

Bardet-Biedl syndrome (BBS) is a pleiotropic disorder comprising blindness, polydactyly, learning disability, obesity, hypogonadism and kidney defects (Forsythe and Beales, 2013). It is one of a growing class of diseases known as ciliopathies. It is a rare condition, affecting 1:100,000 in North America and Europe and is inherited in an autosomal recessive manner (Forsythe et al., 2018).

The over 20 BBS-associated genes encode proteins that regulate the function of primary cilia, microtubule-based structures that project from the cell body (Heon et al., 2016; Priya et al., 2016; Forsythe et al., 2018; McConnachie et al., 2021). Primary cilia have a chemosensory role and host signalling components of the Hedgehog (Hh), Wnt and other growth factor pathways (Corbit et al., 2005; Simons et al., 2005; Rohatgi et al., 2007; Lancaster et al., 2011; Reiter and Leroux, 2017; Elliott and Brugmann, 2019).

BBS proteins help coordinate traffic into and out of the primary cilium. BBS1, 2, 4, 5, 7, 8, 9 and 18 form a complex termed the “BBSome” which has been implicated in vesicular protein transport to the cilium (Nachury et al., 2007; Loktev et al., 2008). The BBSome also interacts with intraflagella transport (IFT) machinery and assists in the ciliary exit of signalling proteins (Zhang et al., 2012a; Eguether et al., 2014; Nozaki et al., 2018). Another three BBS proteins, BBS6, 10 and 12, assist in the assembly of the BBSome (Zhang et al., 2012b), whilst the remaining BBS factors support the composition and function of the primary cilium (Locke et al., 2009; Seo et al., 2011; Estrada-Cuzcano et al., 2012; Aldahmesh et al., 2014; Heon et al., 2016; Schaefer et al., 2016).

In the nephron, primary cilia have a mechanosensory role, bending upon fluid flow and facilitating calcium influx (Praetorius and Spring, 2001). Renal abnormalities are a recurrent feature in ciliopathies (Reiter and Leroux, 2017). Renal disease is the leading factor contributing to early mortality in BBS patients (Riise, 1996), but it is phenotypically variable. A recent study of a United Kingdom population found that 41% of BBS patients (131/322) had chronic kidney disease (CKD), which ranged from mild to severe (Forsythe et al., 2017; 2018). Furthermore, where ultrasound information was available, 51% (90/177) of patients studied had a structural kidney deformity. Defects include developmental abnormalities, such as agenesis, horseshoe, ectopic and duplex kidney, as well as cysts, dysplasia, hydronephrosis, scarring and atrophy (Forsythe et al., 2017; 2018). The reasons why BBS mutations lead to kidney disease are not well understood.

The Human Induced Pluripotent Stem Cell Initiative (HipSci) has established a high quality bank of iPSCs from over 500 healthy and rare disease donors, including lines from patients with mutations in *BBS1*, *2* and *10* (www.hipsci.org) (Kilpinen et al., 2017). Recent advances in directed differentiation of iPSCs have enabled the generation of sophisticated multicellular 3D models that mimic the cellular compositions of human organs, including kidney organoids that contain nephron-like structures with glomeruli, proximal tubules, loops of Henle, distal tubules and collecting ducts (Morizane et al., 2015; Takasato et al., 2015; Taguchi and Nishinakamura, 2017). Mouse BBS mutants fail to accurately phenocopy human BBS kidney disease (Davis et al., 2007; Rahmouni et al., 2008; Marion et al., 2012; Cognard et al., 2015). Therefore, we sought to compare iPSC-derived kidney progenitors and organoids from BBS patients and healthy donors, to uncover defects that could provide insights into the human disease.

## Results

### Phenotypic analysis of kidney progenitor cells

We selected 14 cell lines from healthy donors and 14 lines from BBS patients from the HipSci resource (Supplementary Table S1, S2). The BBS lines comprised 6 from patients with *BBS1* mutations, 5 from patients with *BBS2* mutations and 3 from patients with *BBS10* mutations. Patients with mutations in the same gene did not always have the same DNA variant (Supplementary Table S2). The lines were from male and female donors and covered a similar age range (Supplementary Table S1). All had passed HipSci quality controls, as previously described (Kilpinen et al., 2017).

**FIGURE 1:**
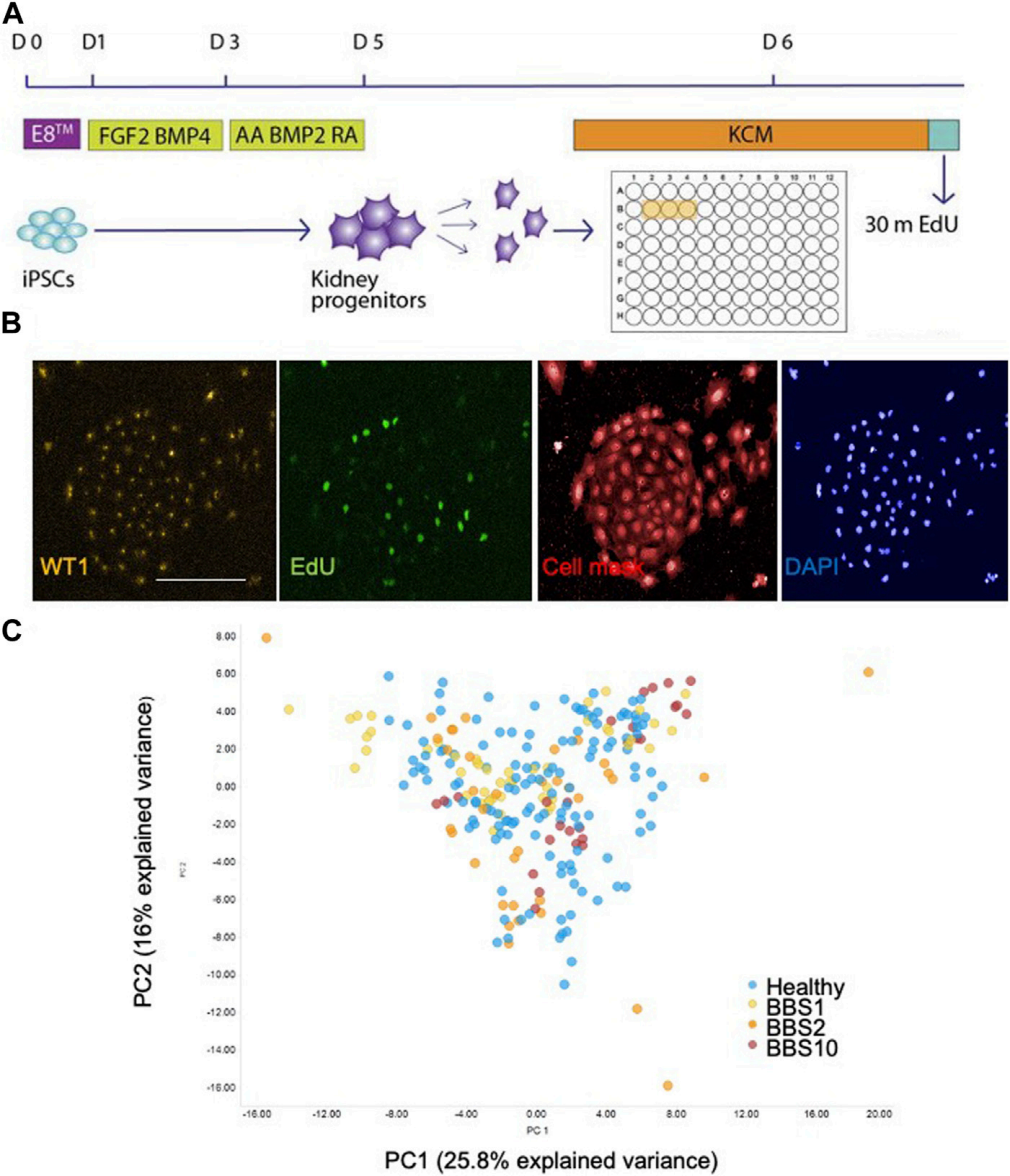
Single cell analysis of kidney progenitor cells. **(A)** Assay set-up. Following differentiation, cells were disaggregated and plated in triplicate wells. Cells are pulsed with EdU prior to fixation. **(B)** Examples of input images of control kidney progenitors for analysis. Scale bar, 300 μm. **(C)** 14 features were recorded per cell. Additional measurements were performed per well, giving a total of 85 data components. PCA was performed on the 85 features. The first two principal components (PC) are plotted, which together explain 41.8% of the variance. 28 donor lines were analysed, comprising 14 lines from healthy donors, 6 *BBS1*, 5 *BBS2*, 3 *BBS10* lines. Data are from three biological replicates per line, each comprising 3 technical replicates (wells). Each data point in the plot corresponds to one well, in which 3,000 cells were plated.

We first compared the properties of kidney progenitors from healthy and BBS donors. iPSCs were differentiated using a previously published protocol in which iPSC colonies were treated with FGF2 and BMP-4 for 2 days followed by retinoic acid, Activin A and BMP2 for 2 days (Figure 1A) (Xia et al., 2013). The following day cells were harvested, replated in 96-well plates, pulse labelled with EdU and fixed 24 h after plating. Differentiation was characterised by a change in morphology (Supplementary Figure S1A), downregulation of the pluripotency marker Nanog, and expression of kidney marker genes, including WT1 (Supplementary Figure S1B).

Fixed cells were labelled with anti-EDU to detect proliferating cells and an antibody to WT1 as a kidney progenitor marker (Xia et al., 2013). Cells were stained with Cell Mask and DAPI to visualise cell shape and nuclear shape, respectively (Figure 1B). Individual iPSC lines were plated in triplicate wells per plate and each experiment was performed three times with different batches of the same cell lines. The plates were subjected to high-content imaging and image analysis software was used to measure a total of 14 features per cell (Supplementary Table S3). For each well, the mean, standard deviation, sum, maximum, minimum, median values and the total number of nuclei were calculated, resulting in 85 data components per well (Supplementary Table S3).

Principal component analysis (PCA) of the 85 features from the lines showed that the healthy and diseased cohorts overlapped almost completely (Figure 1C). To investigate potential genotype-phenotype correlations, we performed multivariate logistic regression. Overall, association testing between Healthy and *BBS1* (Supplementary Table S4) or *BBS2* (Supplementary Table S5) or *BBS10* (Supplementary Table S6) revealed no statistically significant association with phenotypic features, except for DAPI intensity between Healthy and BBS10 lines, where *p* = 0.049 (Supplementary Table S6). We conclude that the BBS mutations did not have a major impact on the capacity of cells to differentiate into WT1-positive kidney progenitors, nor on cell proliferation and morphology under the conditions of the assay.

### Validation of kidney organoid formation by cells from healthy donors

**FIGURE 2:**
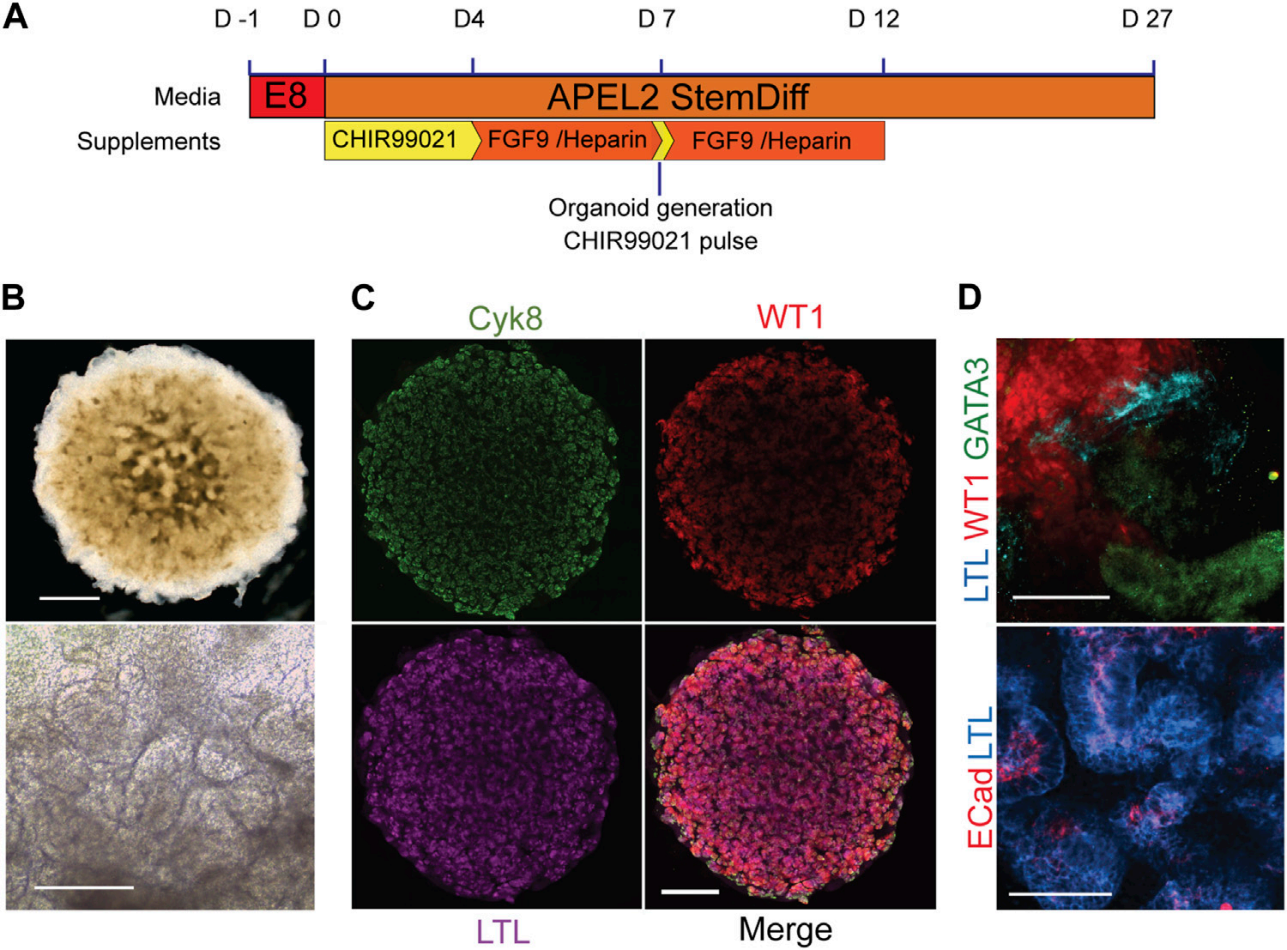
Kidney organoids from healthy donors. **(A)** Schematic of organoid differentiation protocol. **(B)** Representative bright field image of day 20 organoid from a healthy donor (Kegd_2). Top panel scale bar, 1 mm. Lower panel is a higher magnification showing internal architecture. Scale bar, 100 μm. **(C)** Immunofluorescence and lectin labelling of whole organoids for markers of collecting ducts (Cyk8), proximal tubules (LTL) and podocytes (WT1). Scale bar, 1 mm. **(D)** Higher magnification images show segmented nephron compartments: collecting duct (GATA3), podocytes (WT1) and proximal tubules (LTL and ECad). Scale bars, 100 μm.

Having established that there was no significant difference in the ability of healthy, BBS1, BBS2 and BBS10 mutant lines to form WT1-positive progenitors we next examined differentiation into 3D kidney organoids. Cells were subjected to a previously published protocol, adapted to feeder-free culture conditions (Figure 2A) (Takasato et al., 2015; Forbes et al., 2018). We initially differentiated four healthy donor lines (Cuhk_1, Kegd_2, Kute_4 and Hoik_1). The differentiation protocol was performed at least three times on each line and a minimum of three organoids were observed per line per differentiation round. In every case, by day 20 organoids had reached ∼6 mm in diameter and had undergone morphological differentiation, as evidenced by the presence of tubular structures (Figure 2B). The organoids comprised cells that expressed markers of multiple kidney lineages, including WT1-positive podocytes, Lotus Tetragonolubus Lectin (LTL) and E-Cadherin-positive proximal tubules, and GATA3 and Cytokeratin 8 (Cyk8)-positive collecting ducts (Figures 2C, D). The upregulation of kidney marker gene expression was confirmed by Q-PCR (Supplementary Figure S2).

Transmission electron microscopy was used to examine kidney structures at higher resolution (Churg and Grishm, 1975). Tubules were easily identifiable and in cases where the lumen was open, the putative distal and proximal tubules could be distinguished (Supplementary Figure S3). Distal tubules contained sparse short microvilli whilst proximal tubules contained denser, longer villi projections reminiscent of a brush border (Supplementary Figure S3A). Podocyte islands identified glomerular regions, which were surrounded by a membrane that resembled a Bowman’s capsule. The podocytes demonstrated characteristic primary and secondary foot processes (Supplementary Figure S3B).

### Generation of kidney organoids by BBS10 mutant iPSC lines

To examine the potential effect of BBS mutations on kidney organoid formation we focused on *BBS10*. *BBS10* mutations are associated with the most severe renal BBS phenotypes (Imhoff et al., 2011; Esposito et al., 2017; Forsythe et al., 2017) and over 20 pathogenic variants have been reported in the United Kingdom (Forsythe et al., 2017). Three BBS10 patient lines were analysed (Laig, Xiry and Nolz). In the case of Laig and Xiry two independent clonal lines were available (Laig_1, Laig_2 and Xiry_1, Xiry_5 respectively). Each patient line harboured different compound heterozygous variants, but each had one missense and one truncating mutation. DNA and predicted protein changes are illustrated in Supplementary Figure S6. Nolz had the earliest truncating mutation, c.55G>T/c.590A>G (p.Glu19X/p.Tyr197Cys), followed by Xiry, c.235dup/c.989T>C (p.Thr79Asnfs*17/p.Val330Ala) and finally Laig, c.285A < T/c.2119_2120del (p.Arg95Ser/p.Val707X). Patient Xiry presented with late-stage chronic kidney disease (CKD) and had a sibling with mutant BBS10 and end stage renal failure (ESRF). Patient Laig had a recurrent urinary tract infection (UTI) but otherwise normal renal function. The kidney phenotype of Nolz could not be ascertained.

**FIGURE 3:**
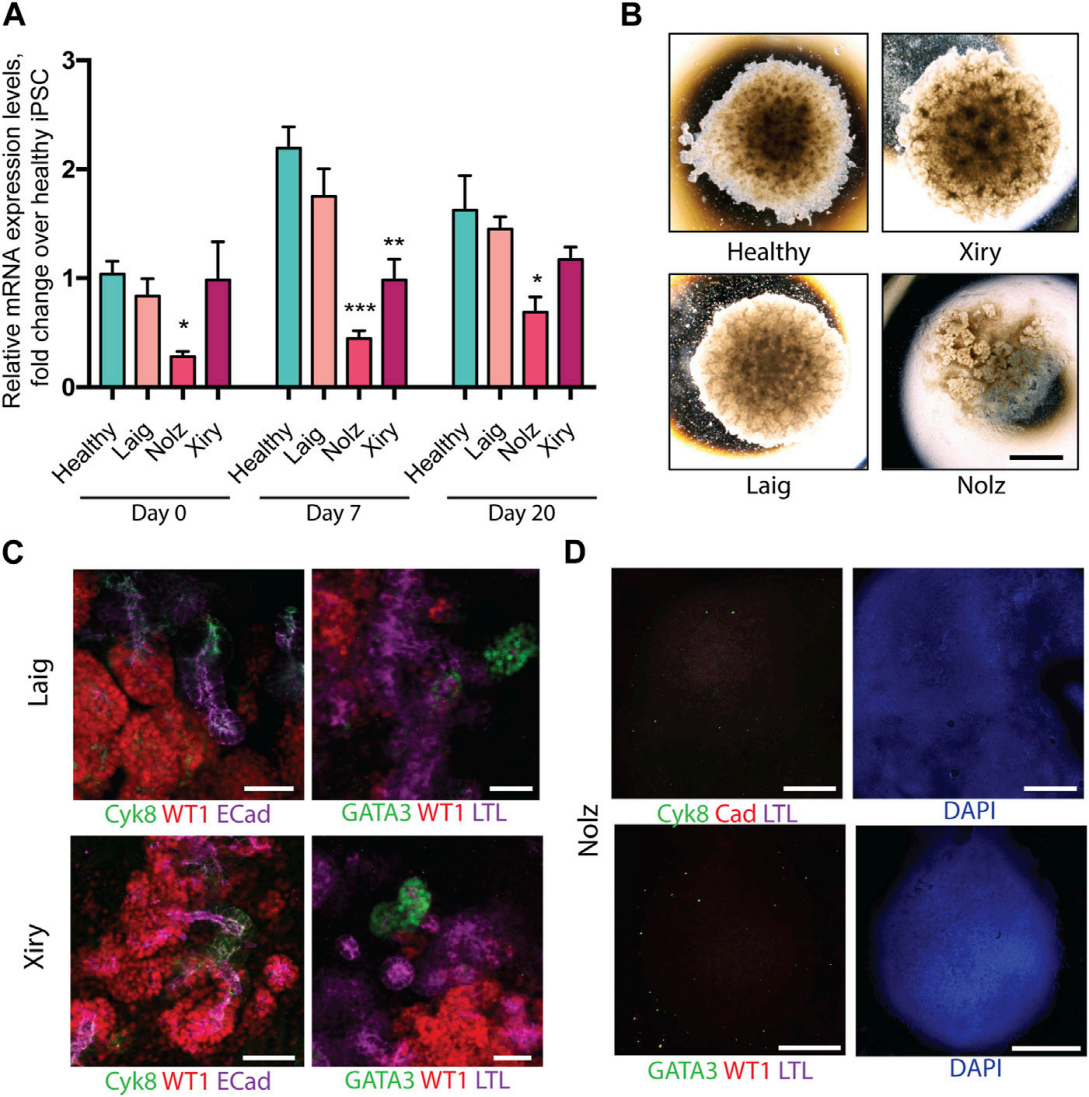
Kidney organoids generated by *BBS10* mutant cell lines. **(A)** Q-PCR of *BBS10* mRNA levels in healthy and mutant lines during differentiation. Healthy data were pooled from three donor lines (Cuhk_1, Hoik_1, Kegd_2) and day 0 contains an additional healthy donor line (Bima_2). For mutant lines *n* = 3–5 independent experiments for each condition and data from Laig and Xiry are pooled from both of their clones. Samples were normalized to GAPDH and 18 S. Primers attached to the 3′ end of the transcript and did not anneal directly over any mutation. Data show mean values ± SEM (one way ANOVA with Dunnet’s *post hoc* test). *p < 0.05, **p < 0.01, ***p < 0.001. **(B)** Bright field images of organoids on day 20. Note abnormal Nolz_4 organoid, with irregular structures, flattened regions and lack of rounded, domed architecture. **(C)** Immunofluorescence labelling of segmented nephron structures in Laig and Xiry organoids on day 20 of culture. **(D)** Immunofluorescence labelling of Nolz_4 organoids on day 20 showing lack of expression of nephron markers. Scale bars: **(B)** 1.5 mm, **(C)** 50 μm, **(D)** 1 mm.

As in the case of the healthy lines, the patient lines were subjected to a minimum of three independent rounds of differentiation and at least three organoids per line per differentiation protocol were analysed. One healthy line was included in each differentiation round as a control. Q-PCR was used to measure *BBS10* mRNA levels during organoid differentiation of healthy and patient lines (Figure 3A). The primers amplified a region towards the 3’ end of the gene, after the mutational sites, not attaching directly over any of the DNA variants. In the pluripotent state (day 0), Laig and Xiry had normal levels of *BBS10* transcripts, whereas Nolz_4 had significantly lower levels (*p* = 0.027; Figure 3A). At day 7, the level of *BBS10* in healthy cells had increased significantly (*p* < 0.001). Levels in Laig also increased and were not statistically different from the healthy lines. However, Nolz_4 and Xiry levels were lower (*p* = 0.0023 and *p* = 0.004, respectively). At day 20, gene expression in healthy cells was not significantly different from day 7. When the *BBS10* lines were compared to healthy lines at day 20, Nolz_4 was the only line to have lower *BBS10* expression (*p* = 0.0385). Thus, in the three lines studied, the earlier the truncating mutation in the *BBS10* gene, the lower the level of mRNA, which is potentially the result of non-sense mediated decay.

Kidney marker gene expression was analysed by Q-PCR at day 0, 7 and 20 of organoid formation (Supplementary Figure S4). None of the BBS10 mutant lines showed a delay in upregulation of gene expression or a reduction in gene expression levels compared to the healthy lines. However, at day 20 the Nolz line exhibited higher expression of the intermediate mesoderm marker OSR1 while the Laig line had higher expression of the metanephric mesoderm marker HOXD11 than the other lines. Brightfield images captured on day 20 of differentiation showed Laig and Xiry formed morphologically normal organoids, with a rounded, domed shape, which were comparable in size to organoids from healthy lines (Figure 3B). In contrast, Nolz_4 organoids were flattened and formed irregular shapes (Figure 3B). Antibody staining showed that Laig and Xiry formed structures that expressed WT1, ECAD, Cyk8 and GATA3 (Figure 3C). In addition the organoids contained LTL-positive structures (Figure 3C). In contrast the Nolz_4 organoids were negative for these markers (Figure 3D). We conclude that while two of the BBS10 lines formed normal organoids, the line with the most severe mutation (Nolz_4) did not.

### BBS10 mutations impact cilia formation

**FIGURE 4:**
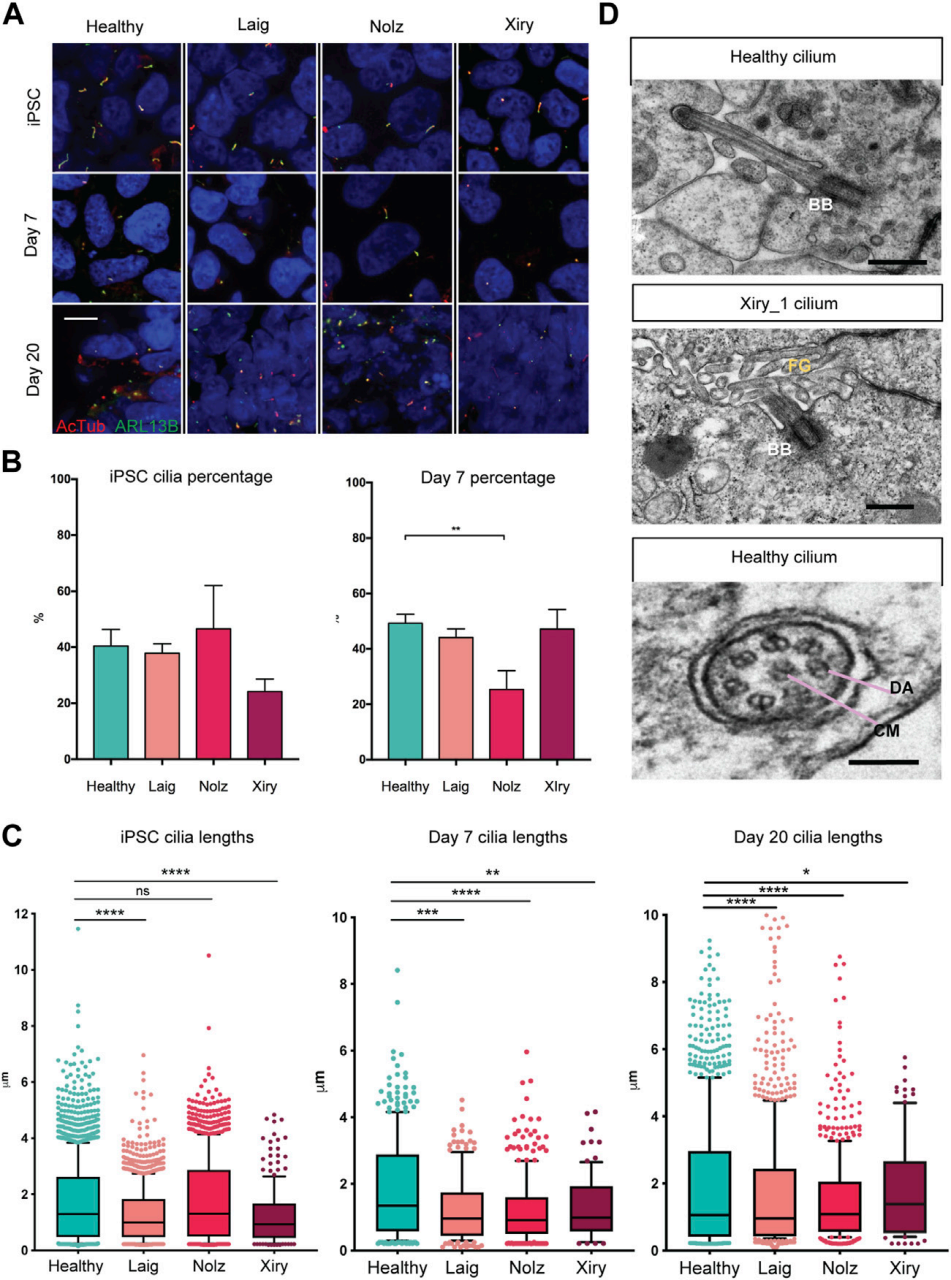
*BBS10* mutations impact cilia length and quantity. **(A)** Immunofluorescence images of primary cilia labelled with antibodies to acetylated tubulin (AcTub) and ARL13B. Undifferentiated (iPSC) and day 7 differentiated cells were grown on glass coverslips. Day 20 images are 18 μm cryosections of organoids. Scale bar, 10 μm. **(B)** Percentage of undifferentiated and day 7 differentiated cells possessing a cilium. 200–1,500 cells were scored per condition over a minimum of three independent experiments. Healthy data are combined from three lines (Kegd_2, Kute_4 and Hoik_1). Data show the mean values ± SEM (one-way ANOVA with Dunnett’s *post hoc* test). **(C)** Box and whisker plots of ciliary lengths in undifferentiated iPSCs and day 7 and day 20 differentiated cells. Each point represents one cilium. Data were pooled from 3–5 independent experiments per line. N = approximately 100 data points (Xiry), > 500 (Healthy, Laig, Nolz). Black horizontal bars show median values. A Kolmoglorov-Smirnov test was used to compare distributions between lines from healthy (Kegd_2, Kute_4 and Hoik_1) and BBS10 donors. *p < 0.05, **p < 0.01, ***p < 0.001, ****p < 0.0001, ns: not significant. **(D)** Transmission electron micrographs of cilia in day 20 organoids. Xiry_1 *BBS10* mutant line showed normal organization of the basal body (BB) and axoneme projecting into luminal, microvilli (MV) filled space. Bottom image, cilium characterized by a central microtubule (CM) pair, and dynein arms (DA), from a healthy organoid (Kegd_2). Scale bars, 500 nm (upper two images), 100 nm (bottom image).

Since BBS10 can influence the function of the primary cilium and cilia can modulate a range of cell features including differentiation (Marion et al., 2009; Irigoín and Badano, 2011; Guo et al., 2017), we examined cilia length and numbers in the BBS10 lines. Acetylated Tubulin (AcTub) and ARL13B were used to identify primary cilia on cells in the undifferentiated state and following induction of differentiation for 7 or 20 days in the organoid protocol (Figure 4A). The percentage of cilia did not differ between healthy and mutant lines in the pluripotent state. However, on day 7, Nolz_4 had significantly fewer cells with cilia compared to Laig, Xiry and the healthy lines (*p* = 0.0018) (Figure 4B). Analysis of cilia number at day 20 was not possible due to the dense packing of cells in different orientations.

A Kolmoglorov-Smirnov test was used to compare cilia lengths (Figure 4C). In the pluripotent state, healthy lines had a median length of 1.3 μm whereas Laig and Xiry had a statistically different distribution with smaller medians, 1 μm for Laig and 0.9 μm for Xiry. Nolz was not significantly different from the healthy lines. At day 7, all three BBS10 lines showed statistically different distributions when compared with healthy cells, which maintained a median length of 1.3 μm. The median for Laig was 1 μm, 0.9 μm for Nolz_4 and 1 μm for Xiry. At day 20, all BBS10 lines showed an altered distribution of ciliary lengths compared to healthy lines. The median for healthy was 1.1 μm; for Laig, 1 μm; for Nolz_4, 1 μm; and for Xiry 1.3 μm.

Transmission electron microscopy (TEM) was used to examine the ultrastructure of primary cilia in kidney organoids. The high-resolution nature of TEM and the 3D orientation of cilia meant that only five cilia from two different healthy lines (Cuhk_1 and Kegd_2) were captured and one BBS10 cilium from the Xiry_1 line. An apically localised basal body and microtubule core projecting into the microvilli-filled tubule lumen showed that the Xiry_1 organelle had an apparently normal transition zone (Figure 4D) although more extensive ultrastructural analysis would be required to ascertain the presence or absence of a cilial defect in this cell line. Motile cilia can be distinguished from primary cilia based on the presence of a central microtubule pair and dynein arms (Ishikawa, 2017). Using this criterion we identified a motile cilium (Figure 4D) in a cell from a healthy line. However, further evaluation in the context of tubular lining epithelial cells of kidney organoids would be required to definitively identify motile cilia in healthy and BBS10 mutant lines.

We conclude that although the BBS10 lines were capable of forming primary cilia, there were reductions in the number (Nolz_4) and length of cilia (all three lines).

### BBS10 kidney organoids undergo spontaneous degeneration

**FIGURE 5:**
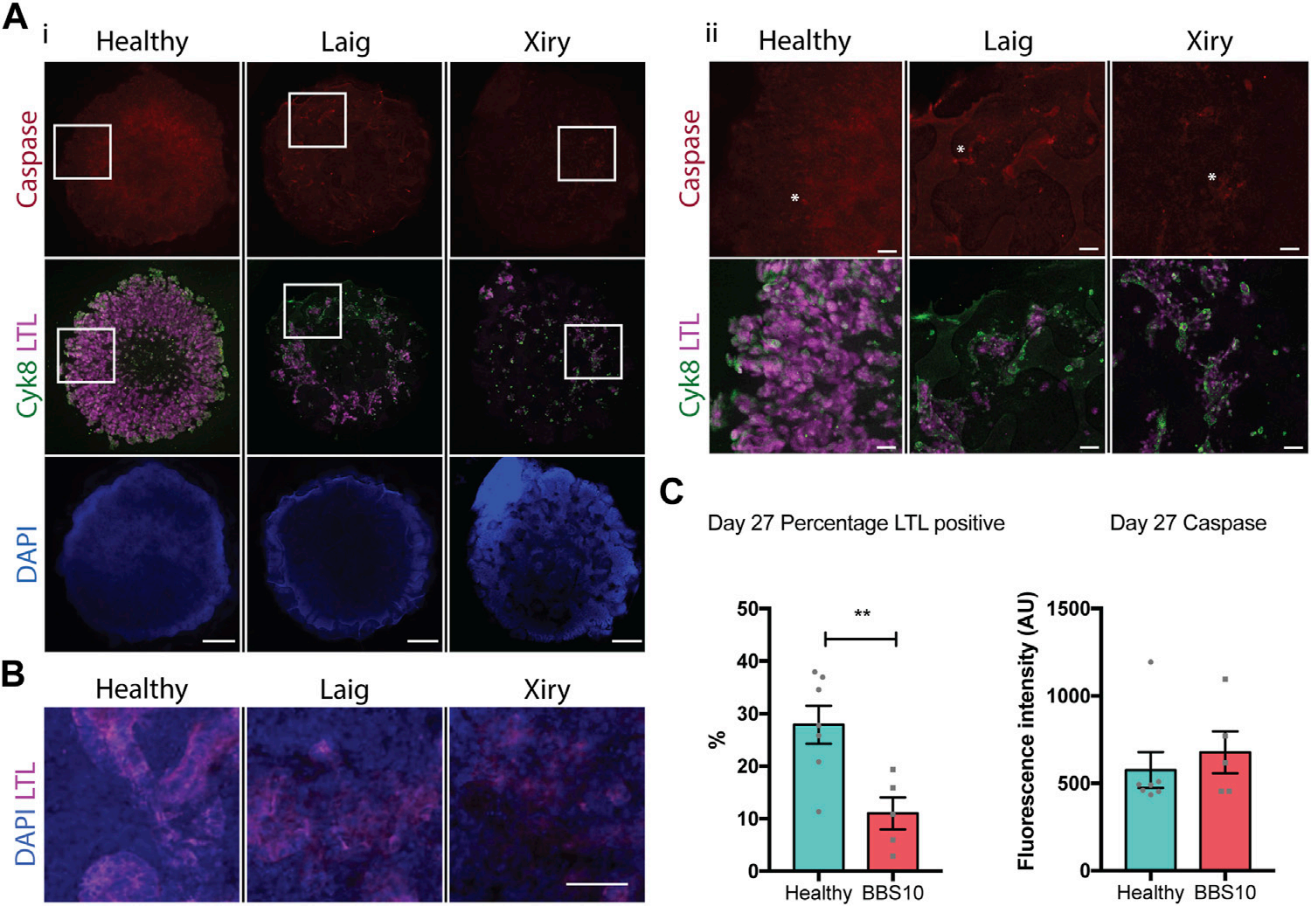
Loss of proximal tubular compartment in *BBS10* mutant kidney organoids. **(A)** Immunofluorescence labelling of day 27 whole organoids. Note reduction in LTL staining of Laig and Xiry lines compared with healthy donor line. Note low intensity of cleaved Caspase-3 in all organoids. Left hand panels (i) show intact organoids. Boxed areas are shown at higher magnification in right hand panels (ii). Asterisks show individual positive cells. **(B)** Higher magnification view of day 27 organoids showing intact tubules in LTL positive regions of healthy organoid, but degeneration in Laig and Xiry organoids. **(C)** Quantification of LTL positive regions and cleaved caspase 3 labelling in whole organoids. % LTL was determined using DAPI to calculate whole organoid volume from multiple Z-plane stacked images. Mean cleaved Caspase-3 intensity per organoid is shown. Each point represents one organoid; each organoid came from an independent experiment. “Healthy” comprises 5 independent differentiations of two donor lines (Hoik_1 and Kegd_2). “BBS10” comprises 5 independent differentiations (Laig x 3 and Xiry x 2). Bars show mean ± SEM with an unpaired Student’s *t* test. **p = <0.01.

While the retina of BBS patients develops normally, the photoreceptors undergo extensive apoptosis and progressive decline, leading to blindness (Mykytyn et al., 2004; Swiderski et al., 2007). Consistent with this, while the BBS10 lines Laig and Xiry showed normal kidney organoid development at 20 days, the organoids showed evidence of degeneration 7 days later, at day 27. Labelling for two epithelial lineages, LTL (proximal tubules) and Cyk8 (collecting ducts), showed that whereas the collecting ducts were normal, there was a marked reduction of the LTL compartment (Figure 5A). At higher magnification, LTL labelling of tubular nephron structures was readily apparent in healthy organoids. In contrast, the structure of tubules in BBS10 organoids was lost and LTL showed diffuse staining (Figure 5B). Our findings suggest that the degeneration of LTL tubules in kidney organoids is a potential *in vitro* surrogate for kidney abnormalities in BBS10 patients.

We examined apoptosis as a potential mechanism of cell death in day 27 organoids. Staining for cleaved Caspase-3 did not reveal any difference between healthy and BBS organoids when the total fluorescence intensity of individual organoids was measured (Figure 5C). Examples of individual positive cells are marked with asterisks in Figure 5A. As a positive control, we treated day 19 organoids with Cisplatin and examined them 24 h later. Cisplatin is nephrotoxic and induces apoptosis as one mechanism of cell death (Yang et al., 2008). Cisplatin treatment led to an increase in cleaved Caspase 3 labelling in Healthy, Laig and Xiry organoids (Supplementary Figure S5). However, there was no statistically significant difference between Healthy and *BBS10* mutant organoids, either at the whole organoid level or within the LTL compartment (Supplementary Figure S5B).

### Confirmation of the BBS10 mutant phenotype

**FIGURE 6:**
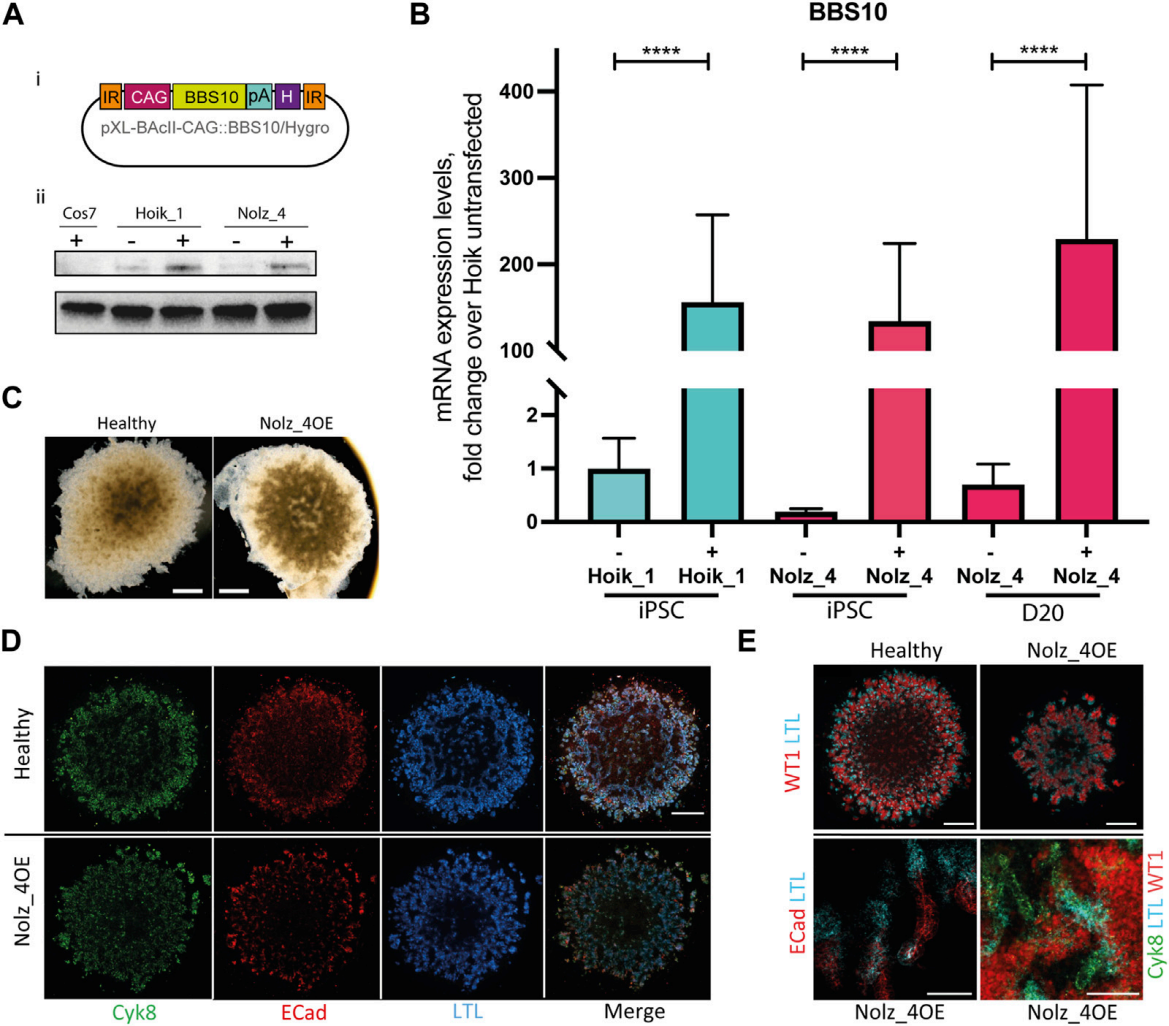
Effects of wild type BBS10 overexpression. **(Ai)** BBS10 plasmid containing the wildtype human *BBS10* gene with a poly Adenylation (pA) tail and Hygromycin (H) resistance gene under the control of a CAGGS promoter. The construct was flanked by inverted repeats (IR) in the pXL-BAcII backbone. **(B)** Q-PCR of *BBS10* mRNA levels without (−) and with (+) the wild type transfected construct in a healthy line (Hoik_1) and patient line (Nolz_4) in pluripotent cells (iPSC) and after differentiation of Nolz_4 at day 20. Three independent experiments for each condition. Samples were normalized to GAPDH and 18 S mRNA. *BBS10* primers attached to the 3′ end of the transcript away from the mutated site. Data show mean values ± SEM (F-test). ****p < 0.0001. **(Aii)** Western blot of untransfected (−) and transfected (+) iPSCs showing upregulation of a band at ∼81 KDa corresponding to BBS10. The monkey fibroblast-like cell line Cos7 was used as a negative control as the BBS10 antibody does not cross-react with monkey proteins. **(C)** Bright field images of day 20 organoids in the healthy Hoik_1 and Nolz_4 over expressing BBS10 (Nolz_4OE). Scale bar, 1 mm. **(D,E)** Day 20 organoids from the healthy line Hoik_1, and patient line overexpressing wildtype *BBS10* (Nolz_4OE) showing labelling for Cyk8, ECad, LTL and WT1. **(E)** Higher magnification of Nolz_4OE in lower panels show presence of markers of nephron structures: proximal tubules (LTL, ECAD) collecting ducts (Cyk8), podocytes (WT1). Scale bars: 1 mm **(D,E)**, 100 μm **(E)**.

To confirm the observed phenotypes were indeed a consequence of mutated *BBS10*, we first used a Piggybac integration system to introduce wild type copies of *BBS10* into one of the patient lines. A construct was generated containing the human *BBS10* gene under the control of the CAG promoter, with a poly Adenylation (pA) signal and a Hygromycin (H) resistance gene, and flanked by inverted repeat (IR) integration sites (Figure 6A). We focussed on Nolz_4 as it demonstrated the most severe phenotype. As a control, we also examined the effects of *BBS10* overexpression on a healthy line, Hoik_1. There was a significant increase in *BBS10* mRNA and protein levels following transfection in both Nolz_4 and Hoik-1 (Figures 6A, B). The faint signal in the Nolz_4 western blot could reflect non-specific antibody labelling, although it is notable that the protein band in untransfected Nolz_4 was lower than in untransfected Hoik_1, consistent with the low levels of mRNA detected by Q-PCR (Figure 3A).

Three rounds of kidney organoid differentiation were performed on the rescued Nolz_4 line (referred to as Nolz_4OE). Bright field imaging showed that Nolz_4OE cells were able to form day 20 organoids of regular shape and size (Figure 6C). Furthermore, fluorescence staining revealed the presence of nephron structures and consistent expression of Cyk8, E-Cadherin and WT1 in rescued samples (Figures 6D, E). LTL labelling was also rescued.

**FIGURE 7:**
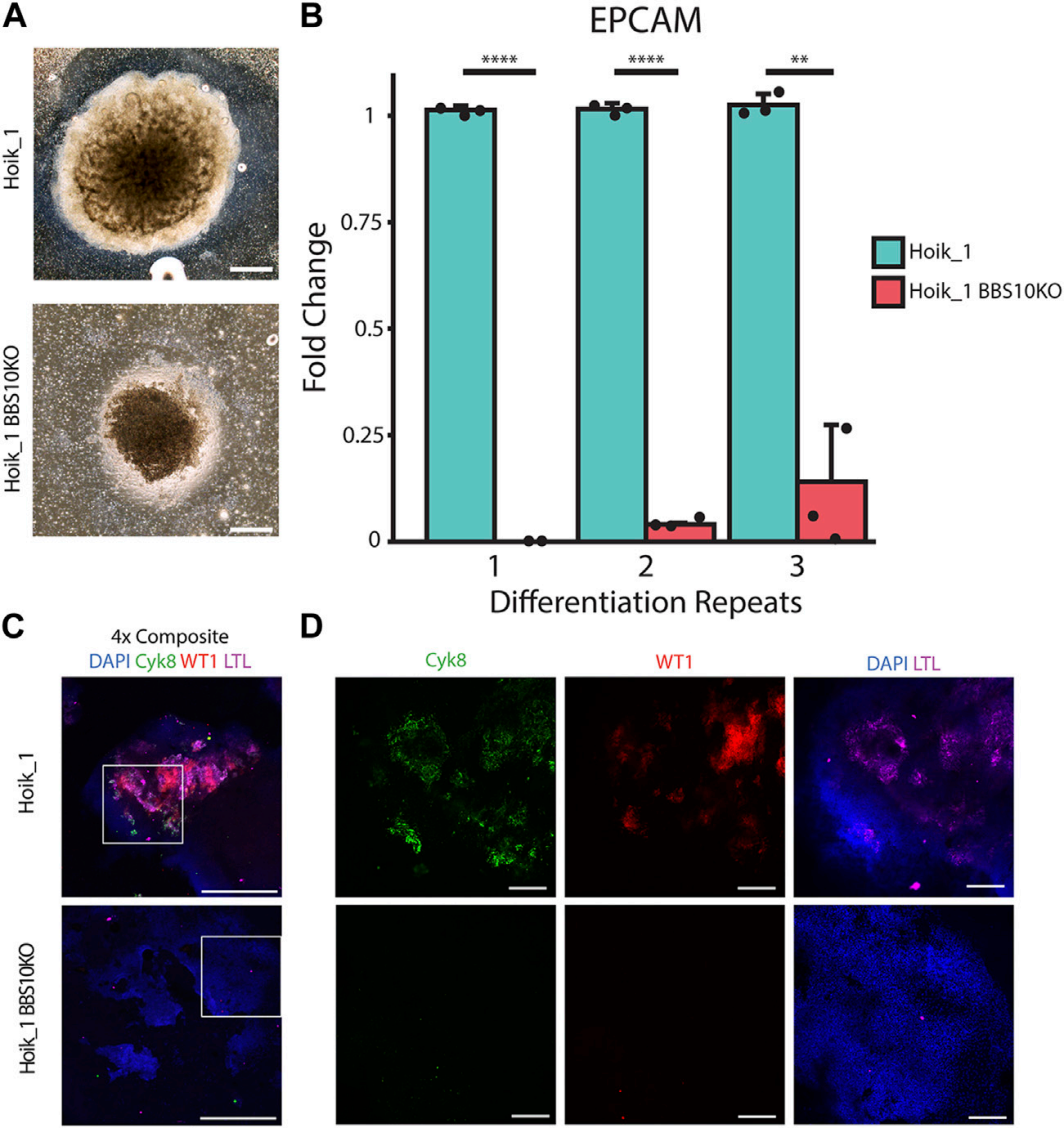
CRISPR induced BBS10 mutation in healthy iPSC results in patient-like phenotypes. **(A)** Brightfield images of day 20 organoids from Hoik_1 and Hoik_1BBS10_KO. **(B)** qPCR analysis of mRNA levels of epithelial marker gene EPCAM in Hoik_1 and Hoik_1BBS10KO_1. Data show mean values ± SEM (Student’s unpaired *t* test). ****p < 0.0001, **p = 0.006. **(C,D)** Confocal images of Hoik_1 and Hoik_1BBS10KO_1 organoids at day 20. CRISPR BBS10KO organoids showed loss of labelling for Cyk8, LTL, and WT1. Boxed regions in **(C)** are shown at higher magnification in **(D)**. Scale bars: 1 mm.

For further confirmation that the kidney organoid phenotypes were attributable to *BBS10* mutations we used CRISPR-Cas9 to generate a BBS10 mutation in the healthy line Hoik_1 via non-homologous end joining (Supplementary Figure S6, Figure 7) (Ran et al., 2013). This allowed us to evaluate the effects of BBS10 mutations in an isogenic background. Sanger sequencing confirmed the presence of a mutation in both alleles of *BBS10*, a one base pair insertion at the Cas9 target site resulting in a frameshift mutation and truncation of BBS10 in a similar position to the Xiry mutation (Supplementary Figure S6).

Three independent rounds of differentiation were performed simultaneously on Hoik_1 and the BBS10KO line. At least three organoids per line per differentiation protocol were examined. After differentiation the morphology of the BBS10KO line was very similar to that seen in Nolz_4, a small organoid which had failed to expand properly, whereas the regular Hoik_1 line was able to grow and differentiate successfully into a large organoid with morphologically visible tubules (Figure 7A). qPCR analysis showed reduced expression of the epithelial marker gene *EPCAM* in BBS10KO cells (Figure 7B). Cyk8, WT1 and LTL labelling revealed positive cells in all healthy Hoik_1 organoids, whereas marker expression in the *BBS10KO* line was not detected (Figure 7C). These observations support an association between *BBS10* truncation and a failure of renal epithelial differentiation within kidney organoids.

## Discussion

The underlying reasons for BBS kidney disease are not well understood. To begin to address this, we utilised iPSC lines from healthy and BBS donors to investigate how BBS mutations impact renal cells. We observed that when a panel of donor lines was differentiated in 2D into WT1-positive kidney progenitors, cellular features, including proliferation, differentiation and cell shape, were indistinguishable between healthy donor lines and lines bearing the mutations in *BBS1*, *2* or *10*. Each of these features has previously been linked to ciliary activity (Irigoín and Badano, 2011; Hernandez-Hernandez et al., 2013; Guo et al., 2017).

*BBS10* is the second most commonly mutated *BBS* gene in the United Kingdom population after *BBS1*, accounting for 20% of patients (Forsythe et al., 2017). We examined three *BBS10* patient lines, all with different compound heterozygous mutations. Varying levels of *BBS10* expression were observed between the lines. This associated with the position of the different truncating mutations: Nolz_4, with the earliest truncation had the lowest expression levels, while Laig had the latest truncation and normal *BBS10* expression levels. The third line, Xiry, with a truncation intermediate in length between the other two lines, exhibited reduced *BBS10* expression at day 7 of differentiation but not at day 20. We speculate that BBS10 mRNA or protein levels may provide a useful tool to predict disease progression. Nevertheless, a limitation of our study is that we did not determine whether both BBS10 alleles (the truncated allele and the missense allele) were expressed in each cell line, and it is therefore possible that there is allele-specific gene silencing.

The Nolz_4 patient line exhibited the lowest level of *BBS10* expression and failed to develop renal structures in organoid culture, a phenotype that was rescued by overexpression of wild type *BBS10*. The organoid defect did not correlate with either a delay in expression of kidney differentiation markers or reduced expression levels compared to healthy organoids. Although information regarding the patient’s kidney function was not available, the *in vitro* phenotype resembles renal agenesis, which is occasionally observed in patients (Beales et al., 1999). Nolz_4 was also the only patient line to have a significant reduction in the number of cells with cilia during differentiation in 2D. Loss of cilia has previously been observed in cultured preadipocytes following *BBS10* knockdown, which led to changes in the activity of downstream Wnt pathway effectors GSKβ and β-catenin (Marion et al., 2009). Wnt signaling plays a key role in renal development and it would therefore be interesting to explore whether the pathway is defective in Nolz_4 cells (Stark et al., 1994; Carroll et al., 2005; Cain et al., 2009; Karner et al., 2011). Analysis of potential changes in Wnt and other signalling pathways in *BBS10* mutant cells would facilitate discovery of the mechanisms underlying defective kidney organoid formation (Low et al., 2019).

Two patient lines, Laig and Xiry, expressed higher levels of *BBS10* mRNA than Nolz_4 and had a milder organoid phenotype. The different kidney lineages formed normally (day 20); however, there was depletion of proximal tubular cells over time as measured by LDL staining (day 27). It would be of interest to determine whether there is a shift in segmentation and patterning of the nephrons as they form within the BBS10 compared to the healthy organoids (Takasato et al., 2015). This would require further quantitative characterisation of additional proximal tubule markers and markers of other nephron segment markers. Our studies also lead us to speculate that the genetic background of individuals with BBS10 mutations could affect any kidney phenotype. This is because when we created an isogenic line with a *BBS10* mutation resembling that of Xiry the cells failed to generate kidney organoids, thus resembling the Nolz line. Analysis of iPSC from healthy donors in the HipSci resource has already highlighted the role of normal genetic variation in influencing cell behaviour *in vitro* (Vickers et al., 2021; Vigilante et al., 2019).

We speculate that the trigger for degeneration in the *BBS10* organoids could be stress induced by hypoxia and sub-optimal nutritional supply as the organoids grew in size (Kelava and Lancaster, 2016; Wörsdörfer and Ergün, 2021). These are physiological consequences that blood vessel damage in diabetes and hypertension, two common secondary features of BBS, can have on kidneys (Griffin, 2017; Libianto et al., 2018). Increased susceptibility to systemic stresses might contribute to kidney functional decline in BBS.

Loss of proximal tubules did not correlate with excessive apoptosis in BBS10 organoids, as assessed by labelling for cleaved Caspase 3. While it would be valuable to measure apoptosis using additional markers such as TUNNEL labelling, it has recently been reported that primary cilia suppress another form of cell death, necroptosis, which could be an alternative potential mechanism for proximal tubule degeneration in the *BBS10* kidney organoids (Kieckhöfer et al., 2022). An alternative driver might be fibrosis since it has been reported that when kidney organoids are maintained for up to 2 months in culture, there is substantial glomerular ECM deposition (van den Berg et al., 2018). Tubulointerstitial fibrosis has been reported in children with BBS, implying it may be a primary feature of the disease (Hurley et al., 1975). Furthermore, proteomic analysis of urine samples from BBS patients has shown an increase in fibrotic proteins compared with healthy controls, even in the absence of a decline in glomerular filtration rate (Caterino et al., 2018). Together these observations suggest further avenues of investigation into the cellular and extracellular changes which occur in patient kidney models.

One interesting observation is that *BBS10* mutant mice only demonstrate severe kidney defects when the gene is knocked out in the whole body and not when it is knocked out specifically in the kidney (Cognard et al., 2015). This is in contrast to our findings, which lead us to speculate that degeneration of LTL tubules in kidney organoids is a potential surrogate for the kidney abnormalities in BBS10 patients. In support of a kidney-autonomous function Seo et al. (2010) found that different *BBS10* mutations in HEK cells had different effects on the interaction with the BBS chaperonin complex, which aids assembly of the BBSome and correct cilia function. The cilia and organoid phenotypes observed in our patient lines may be a consequence of altered interactions with partner BBS proteins. Further analysis of the primary cilium of BBS10 cells would enable investigation of whether specific ciliary and transport proteins are affected. It would also be interesting to discover whether the BBS10 mutation results in other cell lineage-specific effects.

In conclusion, our study has demonstrated that *BBS10* mutations affect kidney organoid formation and maintenance. It will now be of interest to explore the impact of the mutations on specific signalling pathways and the nature of the events that trigger degeneration of the proximal tubules.

## Experimental procedures

### Cell culture

All iPSC lines were generated by the HipSci resource (www.hipsci.org), based at the Wellcome Trust Sanger Institute, United Kingdom. Derivation methods have been previously described (Kilpinen et al., 2017). Quality control checks were used to asses copy number variations, and pluripotency was confirmed based on gene expression profiling (Muller et al., 2011) and antibody staining (Kilpinen et al., 2017). The iPSC lines studied are listed in Supplementary Table S1. Where possible, two clones per donor were analysed. References to donor lines excluding the specific clone number indicate data generated from both clones. Mutations in patient lines were ascertained with gene panel testing of the coding region of 20 BBS-related genes, supplied through the National Health Service BBS clinics. iPSCs were maintained on Vitronectin XF (Stem Cell Technologies) in E8 medium (Invitrogen) with Pen-Strep 1% (v/v) (Sigma-Aldrich) and passaged using Versene (ThermoFisher) every 3–6 days. Cos7 cells were maintained in Dulbecco’s Modified Eagle Medium (ThermoFisher) with 10% fetal bovine serum (FBS) (ThermoFisher) and 1% (v/v) Penicillin-Streptomycin (ThermoFisher).

### Ethics

HipSci lines were generated with appropriate ethics for iPSC derivation (REC Ref: 09/H0304/77, V2 04/01/2013; REC Ref: 09/H0304/77, V3 15/03/2013). BBS samples were collected with separate research ethics approval (REC Ref: 08/H0713/82).

### Generating the piggyBac-BBS10 line

Human BBS10 in the pUC57 plasmid was gifted by Dr Victor Hernandez-Hernandez (University College London, United Kingdom). The coding region of *BBS10* was subcloned into a shuttle vector containing the bovine growth hormone (bGH) poly adenylation (pA) site with a multiple cloning site and ampicillin resistance (Bryson et al., 2014). pUC57-BBS10 was cut with 5′ EcoRI (destroyed during cloning) and 3’ NotI. The shuttle vector was cut with SmaI/NotI and the two fragments were ligated generating a BBS10-pA construct. To assemble the final plasmid, the BBS10-pA fragment was released from its vector with AscI/PacI. The pXL-BAcII-CAG::*Gdnf*/LoxP-Hygro plasmid flanked with piggyBAC terminal repeat sequences is available from Addgene (plasmid number 78209). It was cut with AscI/PacI, removing the *Gdnf* gene. The backbone was ligated with the previously generated BBS10-pA fragment making the final pXL-BAcII-CAG::BBS10pA/LoxP-Hygro construct.

The pXL-BacII-CAG::BBS10pA/LoxP-Hygro construct was transfected into iPSCs with the helper transposase plasmid pCAG::PiggyBac, which has the potential to achieve transposition events in almost all cells receiving the plasmids (Wang et al., 2008). 1 million single iPSCs were resuspended in Opti-MEM (ThermoFisher) and 10 μg of DNA was added, containing both plasmids in a 1:1 ratio. Samples were transferred to cuvettes (NepaGene) and cells were electroporated using the Nepa Super Electroporator NEPA21 instrument. E8 medium containing 10 µM ROCK inhibitor Y27632 was applied to cells immediately following transfection. Cells were subsequently plated on Vitronectin coated plates as previously described. Cells were allowed to recover for 2 days in E8 with 10 µM ROCK inhibitor Y27632, before 50 μg/mL Hygromycin antibiotic (InvivoGen) was used to select for successfully transfected cells over 11 days.

### Generating the BBS10 knockout line

CHOPCHOP (Labun et al., 2019) was used to select 3 different target sites within *BBS10* which were highly ranked and corresponded to different locations within the gene, 2 sites in the first exon where deletions of frameshifts would have the most significant impact on the protein, and 1 site further along in the second exon. RNA oligos corresponding to these sequences (and their complementary sequences) with an extension at each 5’ end corresponding to a BBSI restriction site were ligated, and inserted into the pSpCas9(BB)-2A-GFP (PX458) plasmid after digestion with BBSI. The plasmid was then transformed into bacteria grown on ampicillin plates to select for successfully transformed bacteria. Successful insertion of the gRNA into the plasmid was confirmed via PCR. The pX458 plasmid contains restriction sites for BBSI and Age1. Proper insertion of the gRNA into the plasmid results in the loss of the BBSI restriction site. Digestion with both Age1 and BBSI showed only 1 band, indicating the BBSI site had been lost through successful insertion. Hoik_1 iPSCs were then transfected with this plasmid using Lipofectamine3000 (Invitrogen) and GFP-positive cells were FACS-sorted into separate wells of a culture plate, where they were expanded and banked. Samples of the cells were taken for DNA extraction using DNeasy Blood& Tissue kit (Qiagen), and PCR was performed using primers flanking the Cas9 target site within BBS10. The PCR product was then sent to Source Bioscience for Sanger sequencing.

### Differentiation methods

For the monolayer kidney progenitor assay, differentiation was performed as previously described (Xia et al., 2013). Small iPSC colonies seeded on Matrigel were treated with 50 ng/mL FGF2 (ThermoFisher) and 30 ng/mL BMP-4 (Sigma-Aldrich) for 2 days followed by 1 µM retinoic acid (Sigma-Aldrich), 10 ng/mL Activin A (ThermoFisher) and 100 ng/mL BMP2 (ThermoFisher) for 2 days. The following day cells were detached using Accutase (Biolegend) and plated at a density of 3,000 cells per well on a 96-well, Matrigel-coated plate in triplicate. Cultures were incubated for 23.5 h before a 30 m pulse of 10 µM EdU (ThermoFisher) applied according to the manufacturer’s instructions. Each line was independently differentiated three times.

To generate kidney organoids, differentiation was conducted as previously described (Takasato et al., 2015; 2016) using feeder-free starting conditions (van den Berg et al., 2018), with minor modifications. iPSCs were plated at 19,500/cm^2^, cultured in STEMdiff APEL2 medium (StemCell Technologies) and 5% Protein-free Hybridoma Medium (StemCell Technologies) supplemented with 8 µM CHIR99021 (R&D Systems) for 4 days, followed by 200 ng/mL FGF9 (R&D Systems) and 1 μg/mL heparin for 3 days. Subsequently, 5 × 10^5^ cells were pelleted and each aggregate was transferred to a liquid-air interface culture. After a 1 h pulse of 5 µM CHIR99021, aggregates were cultured with 200 ng/mL FGF9 and 1 μg/mL heparin for 5 days. Growth factors were subsequently withdrawn from the medium and organoids were maintained up to day 27. In some experiments Complete STEMdiff APEL2 medium was supplemented with 5 µM cisplatin (Sigma-Aldrich) at day 19 of differentiation (Takasato et al., 2015) and organoids were cultured for 24 h before fixation and analysis.

### Cilia quantification

For iPSC cilia analysis, cells were passaged as colonies onto vitronectin-coated coverslips and cultured for 3 days to reach approximately 60%–70% confluency. For day 7 samples, differentiation was performed using the protocol of Takasato et al. (2015), adapted to a 24-well plate format with cells seeded on Matrigel coated glass coverslips. For day 20 analysis, samples were differentiated through to the organoid stage, fixed and cryosectioned. Cilia image analysis was performed with ImageJ.

### Immunostaining

For iPSCs, kidney progenitors and day 7 organoids, samples were fixed in 4% PFA for 15 min at room temperature. Samples were washed twice with PBS followed by permeabilisation with 0.1% Triton X-100 (Sigma-Aldrich) and 3% donkey serum (Sigma-Aldrich) in PBS. Where applicable, the Click-iT EdU Alexa Fluor 488 imaging kit (ThermoFisher) was used as directed by the manufacturer. Primary antibodies were incubated over night at 4°C and are detailed in Supplementary Table S7. After 3 PBS washes, species specific secondary antibodies (Alexa Fluor-488 and Alexa Fluor-555, ThermoFisher) were incubated with samples for 1 h. CellMask Deep Red plasma membrane stain (ThermoFisher, 1:1,000) and DAPI (ThermoFisher, 1:1,000) were incubated with cells for 30 min (ThermoFisher). Samples on glass slides were mounted in Prolong Gold anti-fade mountant (ThermoFisher) and samples in 96-well plates were kept in PBS.

For mature organoid wholemount staining, aggregates were fixed from day 20 using an adaptation of a previously described protocol (Takasato et al., 2016). Samples were immersed in 2% (wt/v) PFA, on the membrane, at 4°C for 20 min and washed three times with PBS for 10 min each. Blocking was performed for 2 h in 10% (v/v) donkey serum, 0.06% (v/v) Triton X-100 in PBS and all incubations were performed with gentle rocking. Primary antibodies (Supplementary Table S7) were prepared in blocking buffer and incubated overnight at 4°C with rocking. The following day organoids were washed with PBTX [0.3% (v/v) Triton X-100 in PBS] six times. Species specific secondary antibodies (Alexa Fluor-488, Alexa Fluor-555 and Alexa Fluor-647) were prepared in PBTX at 1:500 dilution and incubated with organoids overnight at 4°C on a rocker. The secondary antibodies were removed and samples were incubated with DAPI in PBS for 3 h followed by three PBS washes and then, still attached to their membranes, were transferred to a glass slide, mounted with hard-set Vectashield medium (Vector Laboratories) and sealed with a coverglass.

### Cryosectioning

Whole organoids fixed in 2% PFA were used for cryosectioning. Samples were incubated in 30% sucrose (Sigma-Aldrich) in PBS for a minimum of 1 h at 4°C. The organoids were subsequently frozen in OCT embedding medium (ThermoFisher) and cut to 18 μm slices using a Thermo Cryostar NX70. Sections were collected on Superfrost glass slides (ThermoFisher). Staining was performed as described for monolayer cultures.

### Imaging and analysis

Brightfield images were captured on an Evos XL core. For the WT1-positive kidney progenitor experiments the Operetta High Content Imaging system (Perkin Elmer) was used for widefield microscopy. Images were captured at a single plane using a 10x objective and the central 9 fields of view were captured in each well of a 96 well plate. Automated image analysis was performed using Harmony Software. All confocal microscopy was performed on an A1 Nikon Upright Confocal Microscope with NIS Elements Software (Nikon). For cilia imaging, a 60x oil immersion objective was used with a 2x digital zoom; optical sections were 0.4 μm apart. Cilia were quantitated with ImageJ by measuring the voxels double positive for ARL13B and Acetylated Tubulin independently by 2-3 observers, one of whom was blinded to experimental conditions. For organoid imaging, 2x and 20x objectives were used with Z-slices. NIS elements software was used to quantify staining in organoids. Z-slices were modelled in 3D and images were then manually thresholded to select positive regions. The DAPI volume was taken as 100% and the LTL positive regions are relative to this.

### Electron microscopy

Kidney organoids were harvested at day 20 for transmission electron microscopy (TEM). Fixation and processing of samples were conducted at the Centre for Ultrastructural Imaging, King’s College London. Organoids on transwell filters were fixed overnight at 4°C with 2.5% (v/v) glutaraldehyde in 0.1 M cacodylate buffer (pH 7.4). After fixation, samples, still on filters, were rinsed multiple times with 0.1 M cacodylate buffer and post-fixed in 1% osmium tetroxide in 0.1 M cacodylate buffer (pH 7.4) for 2 h at room temperature. Samples were then *en bloc* stained with 1% uranyl acetate for 1 h at room temperature, thoroughly washed and dehydrated through a graded ethanol series and infiltrated with epoxy resin (SPURR, Sigma-Aldrich). Finally, organoids were gently detached from the filters, cut into smaller pieces, embedded on flat moulds and polymerised at 70°C for 24 h. Ultrathin sections (70–90 nm) were cut using a Leica UC7 ultramicrotome mounted on 150 mesh copper grids and contrasted using Uranyless (TAAB) and 3% Reynolds Lead citrate (TAAB). Sections were examined at 120 kV on a JEOL JEM-1400Plus TEM fitted with a Ruby digital camera (2 k x2k).

### Western blotting

Cells were lysed with RIPA Buffer (Cell Signalling Technologies) containing PhosSTOP phosphatase inhibitors and Complete mini EDTA-free protease inhibitor cocktail (both Roche). 20 μg of protein was resolved on pre-cast 4%–15% Mini-PROTEAN TGX Gels (Bio-Rad) and then transferred to a PVDF membrane using the Trans-Blot Turbo Instrument and Mini Transfer Packs (both Bio-Rad). Membranes were blocked in 5% skim-milk in Tris-buffered saline with 0.1% Tween 20 (TBS-T) and primary antibodies (Supplementary Table S7) were diluted in blocking buffer and incubated overnight. Membranes were subsequently washed 3 times for 10 min in TBST before incubation with species specific secondary antibody (Peroxidase AffiniPure, Jackson Immuno Research) diluted in TBST, for 2 h at room temperature. A further 3 × 10 min washes were then performed. Clarity ECL Western Blotting Substrate (Bio-Rad) was used to expose antibody reactivity and was applied according to the manufacturer’s guidelines. Imaging was performed using a ChemiDoc Touch instrument (Bio-Rad).

### Q-PCR

RNA extraction was performed using an RNAeasy Mini kit (Qiagen) and 300 ng of RNA was reverse transcribed using a Quantitect Reverse Transcription kit (Qiagen) according to the manufacturer’s instructions. Real-time Q-PCR was performed using the SYBR Green Master Mix (ThermoFischer) with a CFX384 Real-Time System (Bio-Rad). Expression levels were normalized to GAPDH and 18 S values and are shown as fold change relative to the value of the control sample (-ΔΔCT method). A minimum of three biological replicates were used for each condition. Primer sequences are listed in Supplementary Table S8.

## Statistics

Bar graphs show mean ± standard error of the mean (SEM). Unpaired, 2-tailed, Student’s *t* tests, F-test, one-way ANOVA, and Kolmoglorov-Smirnov tests were included where indicated. Dunnet’s *post hoc* tests were used for multiple comparisons. *p* < 0.05 was considered significant in all cases. PCA was performed with TIBCO Spotfire 7.11.1 (Perkin Elmer) and multivariate logistic regression with scripts written for RStudio, Version 1.0.153.

## Data Availability

The original contributions presented in the study are included in the article/Supplementary Material. Further inquiries can be directed to the corresponding author.
