# Supplementary material for: Modelling renal defects in Bardet-Biedl syndrome patients using human iPS cells
Source: Front Cell Dev Biol. 2023 Jun 2;11:1163825. doi: 10.3389/fcell.2023.1163825 (PMC10272764; doi:10.3389/fcell.2023.1163825)
Supplement: Supplementary file 1 [file DataSheet1.pdf]

Supplemental Figures and Tables

Supplemental Figure 1 Characterisation of kidney progenitors. (A) Phase contrast images of control iPSC prior to differentiation (left hand panel) or after differentiation into kidney progenitors (Xia et al., 2013) for 5 days. Scale bar: 200 µm. (B) Q-PCR analysis of mRNA levels of pluripotency (*NANOG*), intermediate mesoderm (*OSR1*, *GATA3*), ureteric bud (*GATA3*, *RET*, *HOXB7*) and metanephric mesenchyme (*GDNF*, *WT1*) markers after five days of differentiation in a healthy line, Bima_2. Samples were from independent experiments and normalized to *GAPDH*. Data show mean values ±SEM with Student's unpaired T-Test * $p < 0.05$, ** $p < 0.01$, *** $p < 0.001$.

Supplementary Figure 2 Characterisation of kidney organoids from healthy donors. Q-PCR analysis of mRNA levels of the markers shown on days 0, 7 and 20 of differentiation. OSR1, intermediate mesoderm. RET, ureteric bud. HOXB7, ureteric bud. GATA3 ureteric bud and collecting duct. HOXD11, metanephric mesenchyme. GDNF, metanephric mesenchyme. Each data point represents one organoid from each of 4 different healthy donor lines (Cuhk_1, Kegd_2, Kute_4 and Hoik_1). Some donor lines were differentiated more than once in independent experiments. Expression levels of the target genes are normalised to GAPDH and 18S. Data show the mean ±SEM with Student's unpaired T-Test. * $p < 0.05$, ** $p < 0.01$, *** $p < 0.001$.

Supplementary Figure 3 Transmission electron microscopy of kidney organoids from healthy donors. (A) Organoids contained different tubule types, which were either closed, contained relatively sparse short microvilli (m) (distal tubule) or densely packed with microvilli characteristic of the brush border (bb) (proximal tubule). (B) Podocytes arranged in clusters surrounded by a basement membrane capsule. Podocytes demonstrated large nuclei and primary (pf) and secondary foot (sf) processes.

Supplementary Figure 4 Q-PCR analysis of kidney markers in healthy and BBS10 organoids. *RET* (ureteric bud), *HOXB7*, (ureteric bud), *OSR1*, (intermediate mesoderm), GATA3 (intermediate mesoderm/ureteric bud/collecting duct), HOXD11, (metanephric mesoderm), GDNF, (metanephric mesoderm), NPHS1, (podocytes). Each data point represents one organoid from an independent experiment. Healthy comprises 4 different donor lines. Expression levels of the target gene are normalised to GAPDH and 18S. Data show mean values and SEM. One way ANOVA with a Tukey's post-hoc test was used at each time point to compare individual BBS10 lines with the healthy line expression. * $P < 0.05$, **** $P < 0.0001$.

Supplementary Figure 5 Effects of Cisplatin on organoid apoptosis. Confocal images stained for LTL+ proximal tubules (green), Cyk8+ collecting ducts (blue), and Cleaved-Caspase 3 (red). Top three panels show day 20 organoids without cisplatin treatment but with sterile water as a control. Bottom two panels show treatment with 5 µM cisplatin for 24 h. Scale bar, 1mm. (B) Intensity of

Cleaved-Caspase 3 staining in whole organoids and in the proximal tubule (LTL+) regions. Fluorescence intensity is shown in arbitrary units (AU), across 3D volume from multiple Z-plane images. Healthy -Cisplatin: n = 7 (Hoik_1 x 2 independent differentiations, x 2 technical replicates. Kegd_2 x 2 independent differentiations, with 2 and 1 technical replicates). BBS10 -Cisplatin: n = 9 (Laig x 3 independent differentiations, with 2, 2 and 1 technical replicates. Xiry x 2 independent differentiations, with 3 and 1 technical replicates). Healthy +Cisplatin: n = 3 (Hoik_1 x 2 independent differentiations, Kegd_2 x 1 independent differentiation). BBS10 +Cisplatin: n = 6 (Laig x 2 independent differentiations with 1 and 3 technical replicates. Xiry x 1 independent differentiation, with 2 technical replicates).

Supplementary Figure 6 Nature of *BBS10* mutations in patient iPSC. (A) The *BBS10* gene showing locations of the DNA mutations in red and Cas9 target sites in green. (B) The BBS10 protein showing the resulting amino acid changes and predicted truncations for patient lines, and the sites at which mutations would occur within the *BBS10k/o* lines. Coloured segments show different domains of the protein Reference transcript ENST00000393262.3, Ensemble.org.

Supplemental Table 1 Healthy donor iPSC lines

HipSci code	Age	F/M
BIMA_2	40-44	M
CUHK_1	45-49	F
EIPL_1	40-44	F
FFDC_11	40-44	M
HOIK_1	40-44	F
JOGF_2	30-34	M
KEGD_2	40-44	M
KUTE_4	25-29	F
OIKD_2	40-44	F
SEHP_2	25-29	F
SITA_1	40-44	M
SOJD_3	45-49	F
TOCO_5	55-59	F
WUYE_3	30-34	F

Supplemental Table 2 BBS donor iPSC lines

HipSci code	Age	F/M	Gene	Mutations 1 and 2
EAVO_1	30-34	F	BBS1	c.1169T>G/p.Met390Arg<br>c.217G>T/p.Gly73X
FOYJ_2	50-54	F	BBS1	c.851del/p.Tyr284Serfs*5<br>c.1169T>G/p.Met390Arg
GIBE_2	35-39	M	BBS1	c.1169T>G/p.Met390Arg<br>c.217G>T/p.Gly73X
JETZ_1	40-44	F	BBS1	c.1169T>G/p.Met390Arg<br>c.47+8C>T
RENG_4	40-44	M	BBS1	c.1169T>G/p.Met390Arg<br>c.1040delT/p.Met347ArgfsX27
WIZE_1	45-49	F	BBS1	c.1169T>G/p.Met390Arg<br>c.1169T>G/p.Met390Arg
EISK_3	30-34	M	BBS2	c.311A>C/pAsp104Ala<br>c.1895G>C/p.Arg632Pro
KECW_3	25-29	F	BBS2	c.311A>C/pAsp104Ala<br>c.1895G>C/p.Arg632Pro
KOJV_1	55-59	F	BBS2	c.823C>T/p.Arg275*<br>c.1286_1287del/p.Val429Glyfs*44
OUZK_4	35-39	M	BBS2	c.1197del/p.His399Glnfs18<br>c.2060-1G>T
VERF_1	20-24	F	BBS2	c.780_782dup/p.Leu260_Ile261insMet<br>c.1572_1575del/p.His525Phefs*21
LAIG_2	25-29	F	BBS10	c.285A<T/p.Arg95Ser<br>c.2119_2120del/p.Val707X
NOLZ_4	25-29	M	BBS10	c.55G>T/p.Glu19X<br>c.590A>G/p.Tyr197Cys
XIRY_5	35-39	M	BBS10	c.235dup/p.Thr79Asnfs*17<br>c.989T>C/p.Val330Ala

Supplemental Table 3 List of 14 features recorded per cell in the high-content image analysis pipeline. For each cell feature, 6 values per well, giving a total of 85 data components captured per well.

Output features per cell

1. Nuclear roundness
2. Nuclear area
3. Nuclear width
4. Nuclear length
5. Ratio of nuclear width : length
6. Dapi Intensity
7. EdU Intensity
8. WT1intensity
9. Cell Area
10. Cell roundness
11. Cell width
12. Cell Length
13. Ratio of nuclear width : length
14. Number of cells per clump

For each feature, the following is calculated

1. Mean per well
2. StdDev per well
3. Sum per well
4. Max per well
5. Min per well
6. Median per well

+ The number of nuclei per well

(14 x 6) +1 = 85 data components per well

Supplemental Table 4 Multivariate logistic regression analysis performed on Healthy vs BBS1 cells.

Feature	Estimate	Std. Error	z value	FDR adjusted p-value
Number of Nuclei - per Well	1.112	1.129	0.984	0.400
Nucleus Area - Mean per Well	-2.904	2.450	-1.185	0.378
Nucleus Roundness - Mean per Well	5.628	2.024	2.781	0.087
Nucleus Width - Mean per Well	-4.582	2.243	-2.043	0.131
Nucleus Length - Mean per Well	6.622	2.972	2.228	0.104
Nucleus Ratio Width to Length	3.306	1.881	1.757	0.200
Edu Intensity - Mean per Well	2.180	1.366	1.596	0.221
DAPI Median - Mean per Well	-4.891	1.973	-2.479	0.104
WT1 intensity - Mean per Well	-0.172	0.856	-0.201	0.840
Cell Area - Mean per Well	4.226	4.236	0.998	0.400
Cell Roundness - Mean per Well	-2.102	1.884	-1.116	0.385
Cell Width - Mean per Well	-2.825	3.404	-0.830	0.451
Cell Length - Mean per Well	-3.843	4.790	-0.802	0.451
Cell Ratio Width to Length - Mean per Well	-2.946	2.024	-1.456	0.259
Cells per clump - Mean per Well	-2.891	1.269	-2.278	0.104

Multivariate logistic regression was performed on 14 features from kidney progenitor cells consisting of lines from 14 healthy donors and 6 BBS1 donors. FDR, false discovery rate.

Supplemental Table 5 Multivariate logistic regression analysis performed on Healthy vs BBS2 cells.

Feature	Estimate	Std. Error	z value	FDR adjusted p-value
Number of Nuclei - per Well	3.682	4.211	0.874	0.470
Nucleus Area - Mean per Well	-1.361	1.341	-1.015	0.414
Nucleus Roundness - Mean per Well	-1.462	2.999	-0.488	0.715
Nucleus Width - Mean per Well	-1.631	1.308	-1.247	0.378
Nucleus Length - Mean per Well	-5.890	3.206	-1.837	0.212
Nucleus Ratio Width to Length	3.777	2.484	1.520	0.294
Edu Intensity - Mean per Well	4.922	2.476	1.988	0.212
DAPI Median - Mean per Well	-1.751	1.378	-1.271	0.378
WT1 intensity - Mean per Well	1.518	0.975	1.556	0.294
Cell Area - Mean per Well	-0.972	0.953	-1.019	0.414
Cell Roundness - Mean per Well	4.178	3.864	1.081	0.414
Cell Width - Mean per Well	-0.653	1.629	-0.401	0.735
Cell Length - Mean per Well	-7.044	3.371	-2.090	0.212
Cell Ratio Width to Length - Mean per Well	0.400	5.001	0.080	0.936
Cells per clump - Mean per Well	3.968	2.065	1.921	0.212

Multivariate logistic regression was performed on 14 features from kidney progenitor cells consisting of lines from 14 healthy donors and 5 BBS2 donors. FDR, false discovery rate.

Supplemental Table 6 Multivariate logistic regression analysis performed on Healthy vs BBS10 cells.

Feature	Estimate	Std. Error	z value	FDR adjusted p-value
Number of Nuclei - per Well	0.399	0.653	0.611	0.752
Nucleus Area - Mean per Well	-0.403	1.433	-0.281	0.831
Nucleus Roundness - Mean per Well	1.058	1.009	1.048	0.524
Nucleus Width - Mean per Well	-1.921	1.659	-1.157	0.494
Nucleus Length - Mean per Well	3.047	1.317	2.313	0.110
Nucleus Ratio Width to Length	1.876	0.966	1.943	0.185
Edu Intensity - Mean per Well	0.032	0.330	0.098	0.922
DAPI Median - Mean per Well	2.087	0.761	2.744	0.049
WT1 intensity - Mean per Well	-1.353	0.713	-1.898	0.185
Cell Area - Mean per Well	6.010	4.124	1.458	0.387
Cell Roundness - Mean per Well	1.429	1.112	1.285	0.454
Cell Width - Mean per Well	-1.252	2.296	-0.545	0.752
Cell Length - Mean per Well	-2.571	3.925	-0.655	0.752
Cell Ratio Width to Length - Mean per Well	-0.592	1.163	-0.509	0.752
Cells per clump - Mean per Well	-0.210	0.597	-0.352	0.829

Multivariate logistic regression was performed on 14 features from kidney progenitor cells consisting of lines from 14 healthy donors and 3 BBS01 donors. FDR, false discovery rate.

Supplemental Table 7 Antibodies and lectin used for fluorescence microscopy (ICC) stains and western blots (WB).

Antibody/ Lectin	Company	Product number	Clone	Species	Use
Acetylated-tubulin antibody	Sigma-Aldrich	T6793	6-11b-1	Mouse mAb	1:500 ICC
ARL13B	Proteintech	17711-1-AP-20	-	Rabbit pAb	1:250 ICC
BBS10	Proteintech	12421-2-AP	-	Rabbit pAb	1:1000 WB
Cleaved Caspase-3	Cell Signalling	9661	5A1E	Rabbit mAB	1:250 ICC
Cytokeratin-8 (TROMA)	DSHB	-	-	Rat mAb	1:250 ICC
E-Cadherin	Produced In-house	-	HECD1	Mouse mAb	1:200 ICC
GAPDH	Abcam	ab9485	-	Rabbit pAb	1:3000 WB
GATA3	Santa Cruz	sc-268	-	Mouse mAb	1:200 ICC
LTL - Biotinylated	Vector Laboratories	B-1325	-	Lectin	1:300 ICC
WT1	Abcam	ab89901	-	Rabbit mAb	1:250 ICC

Supplemental Table 8 Primer list.

	5' Forward primer	3' Reverse primer
18S	GGCCTCACTAAACCATCCAA	GCAATTATTCCCCATGAACG
BBS10	ACCATTACTCAAGAACAATTCC	GTACCCATATTCGGTAACTTAC
EPCAM	AATCGTCAATGCCAGTGTACTT	TCTCATCGCAGTCAGGATCATAA
GAPDH	AGCAATGCCTCCTGCACCACCAAC	CCGGAGGGGCCATCCACAGTCT
GATA3	CGTCCTGTGCGAACTGTCA	GTCCCCATTGGCATTCCTCC
GDNF	CCAACCCAGAGAATTCCAGA	AGCCGCTGCAGTACCTAAAA
HOXB7	CGATGCAGGGCTTGTACCCC	GGCCTCGTTTGCGGTCAGTT
HOXD11	CCACGGTCAACTCGGGACCT	TTCCTACAGACCCCGCCGTG
NPHS1	GAGTATGAGTGCCAGGTCGG	ATGGTGATGTCAGGTGCTGG
OSR1	CTGCCCAACCTGTATGGTTT	CGGCACTTTGGAGAAAGAAG
RET	CTCGACGACATTTGCAAGAA	AGCATTCCGTAGCTGTGCTT
WT1	GCGGAGCCCAATACAGAATA	GATGCCGACCGTACAAGAGT

Figure S1

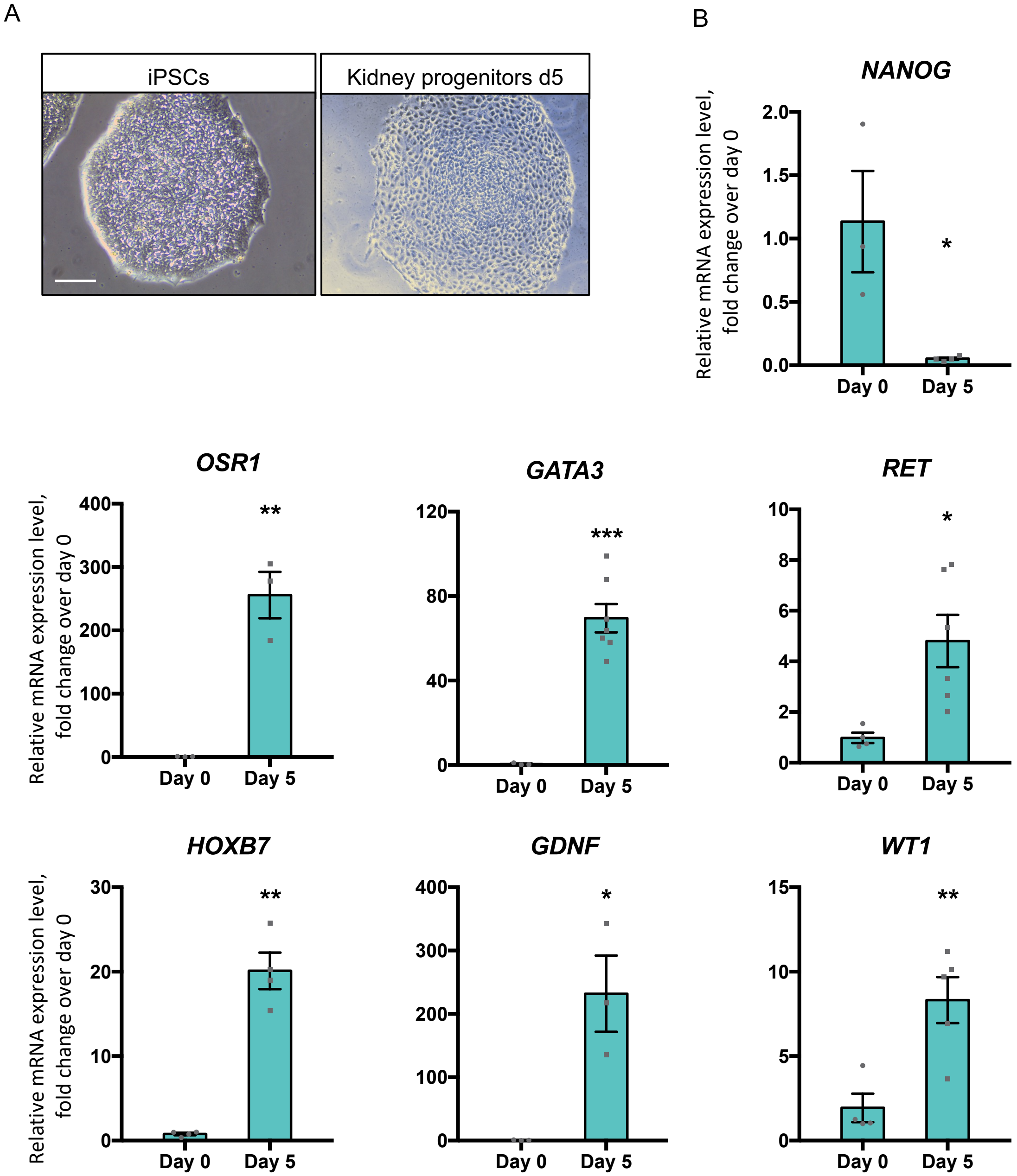
A
iPSCs
Kidney progenitors d5
B
NANOG
Relative mRNA expression level, fold change over day 0
2.0
1.5
1.0
0.5
0.0
*
Day 0
Day 5
OSR1
400
300
200
100
0
**
GATA3
120
80
40
0
***
RET
10
8
6
4
2
0
*
HOXB7
30
20
10
0
**
GDNF
400
300
200
100
0
*
WT1
15
10
5
0
**

Figure S2

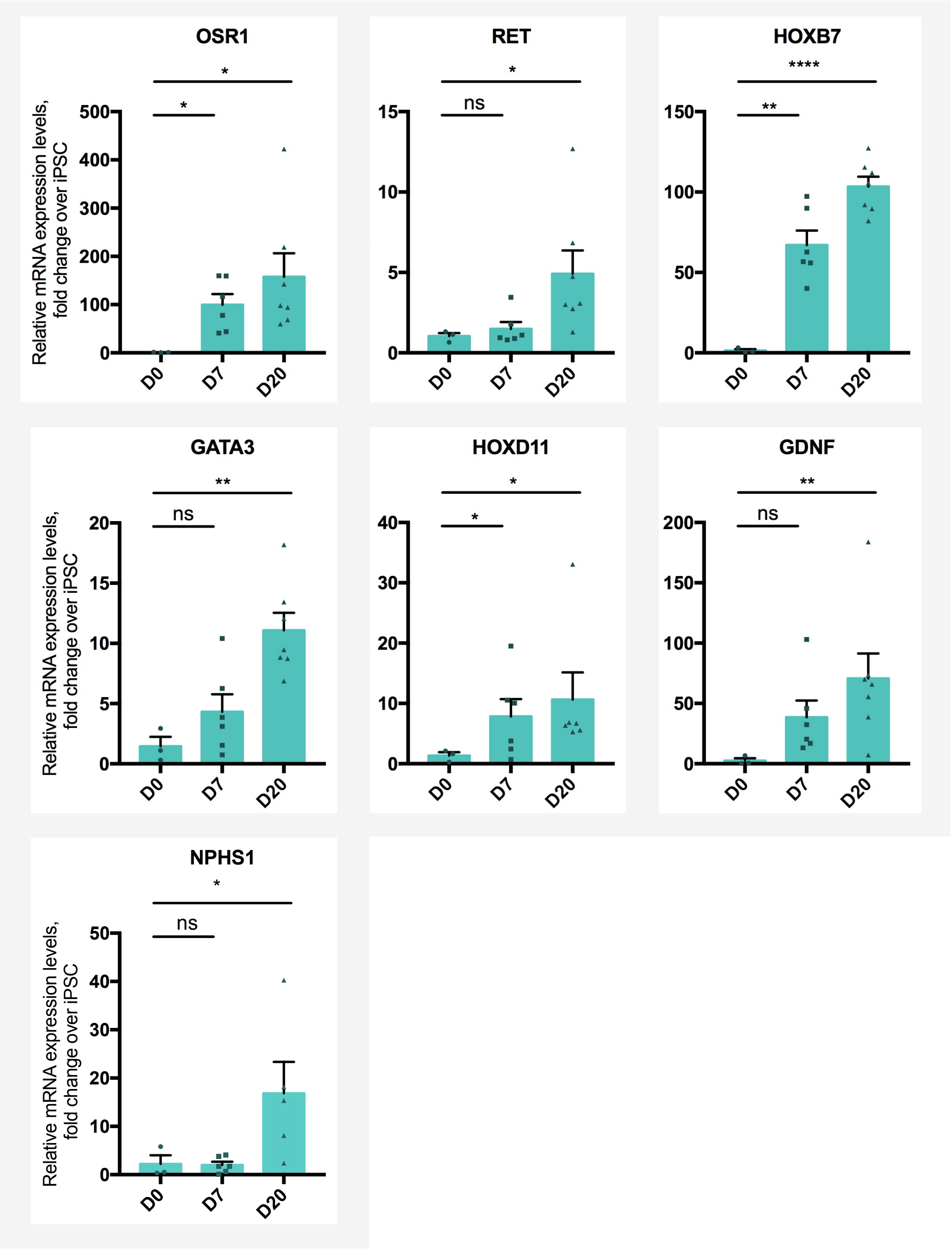
OSR1
RET
HOXB7
GATA3
HOXD11
GDNF
NPHS1
Relative mRNA expression levels, fold change over iPSC
D0
D7
D20
*
**
****
ns

Figure S3

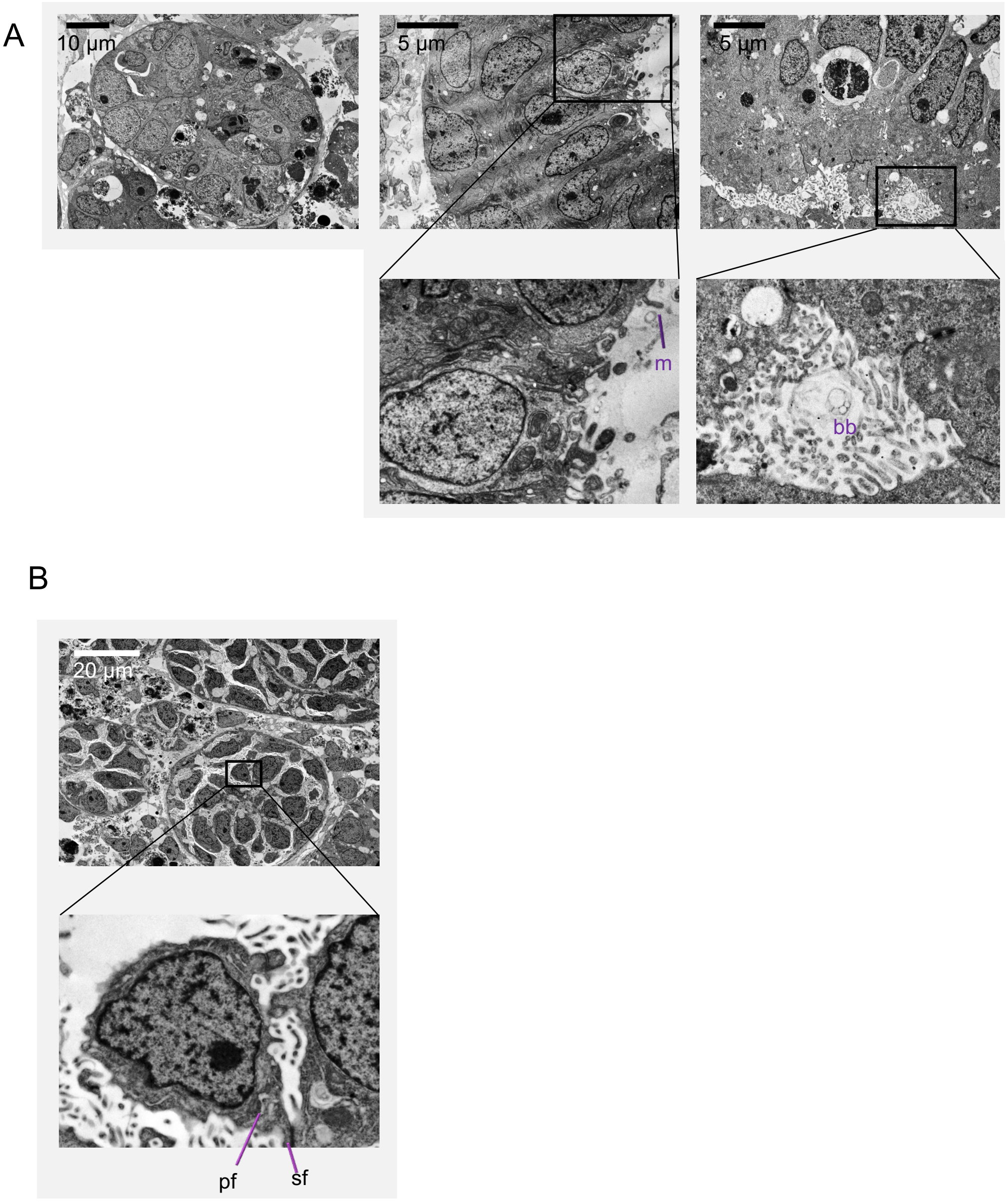

Figure S4

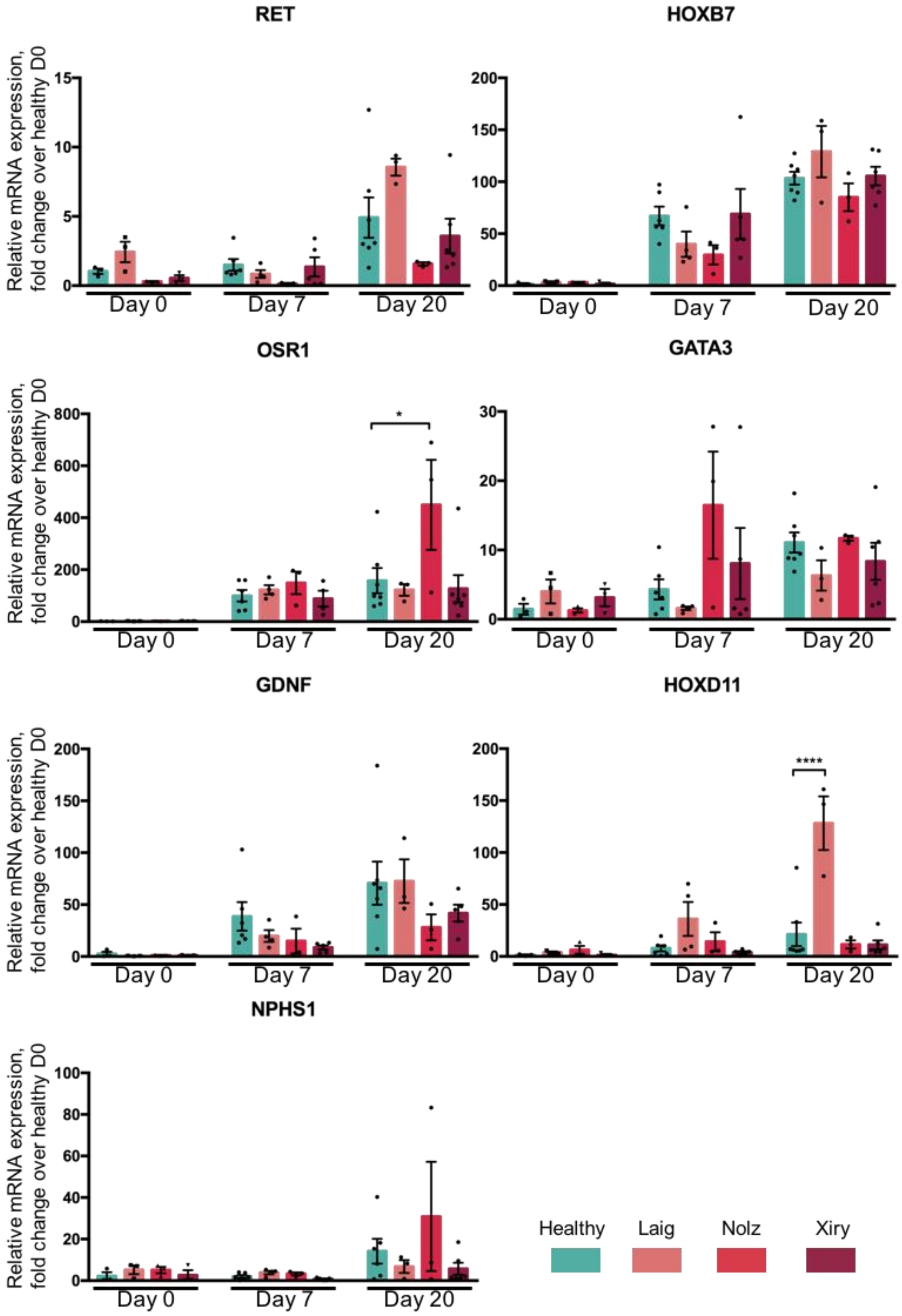

RET
HOXB7
OSR1
GATA3
GDNF
HOXD11
NPHS1
Relative mRNA expression, fold change over healthy D0
Day 0
Day 7
Day 20
*
****
Healthy
Laig
Nolz
Xiry

Figure S5

A

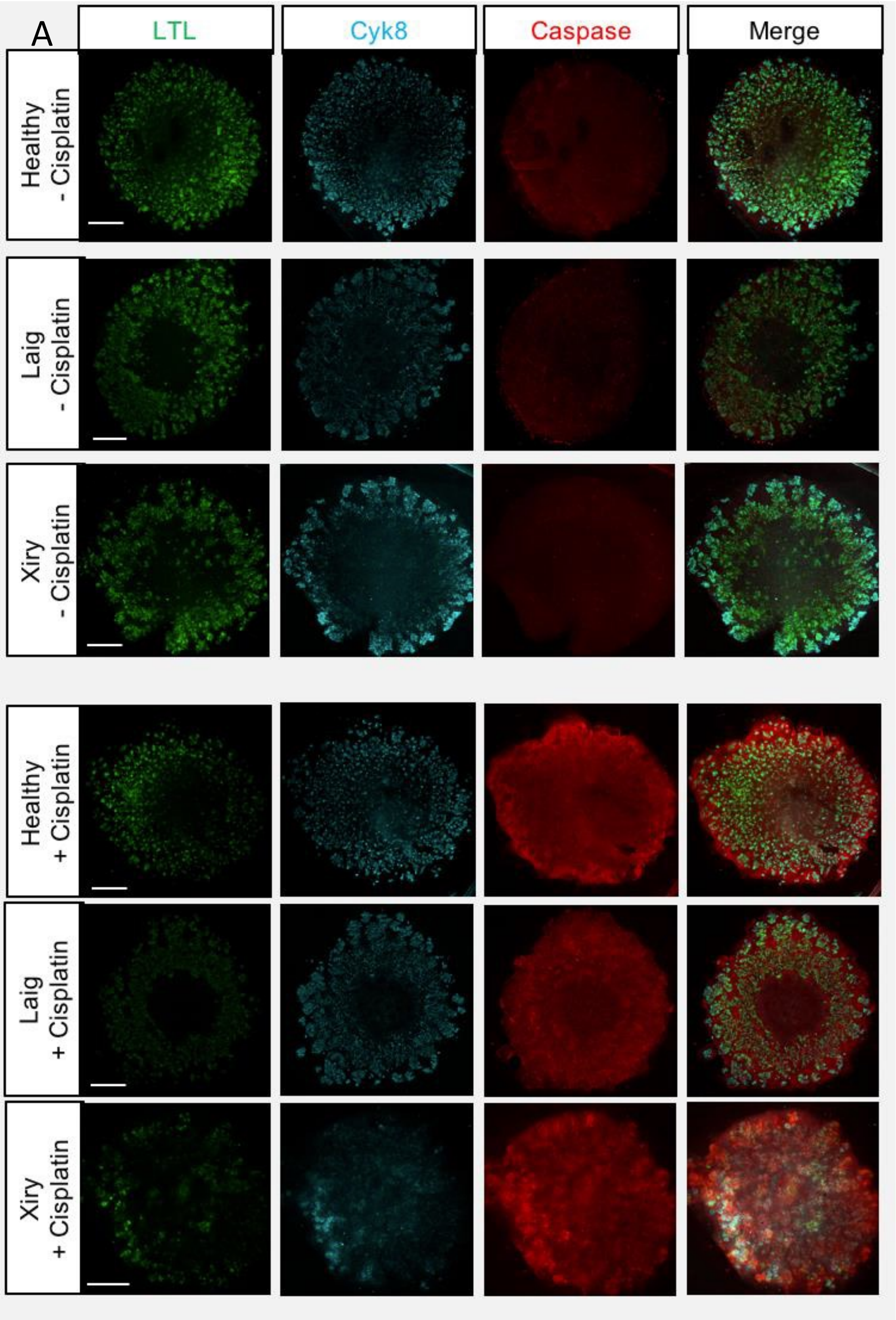
LTL
Cyk8
Caspase
Merge
Healthy - Cisplatin
Laig - Cisplatin
Xiry - Cisplatin
Healthy + Cisplatin
Laig + Cisplatin
Xiry + Cisplatin

B

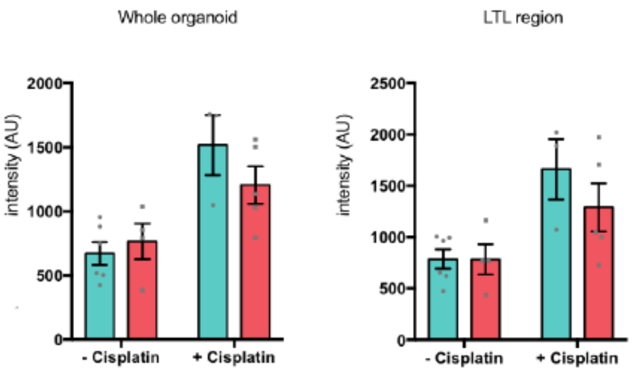
Whole organoid
LTL region
intensity (AU)
2000
1500
1000
500
0
2500
- Cisplatin
+ Cisplatin

Figure S6

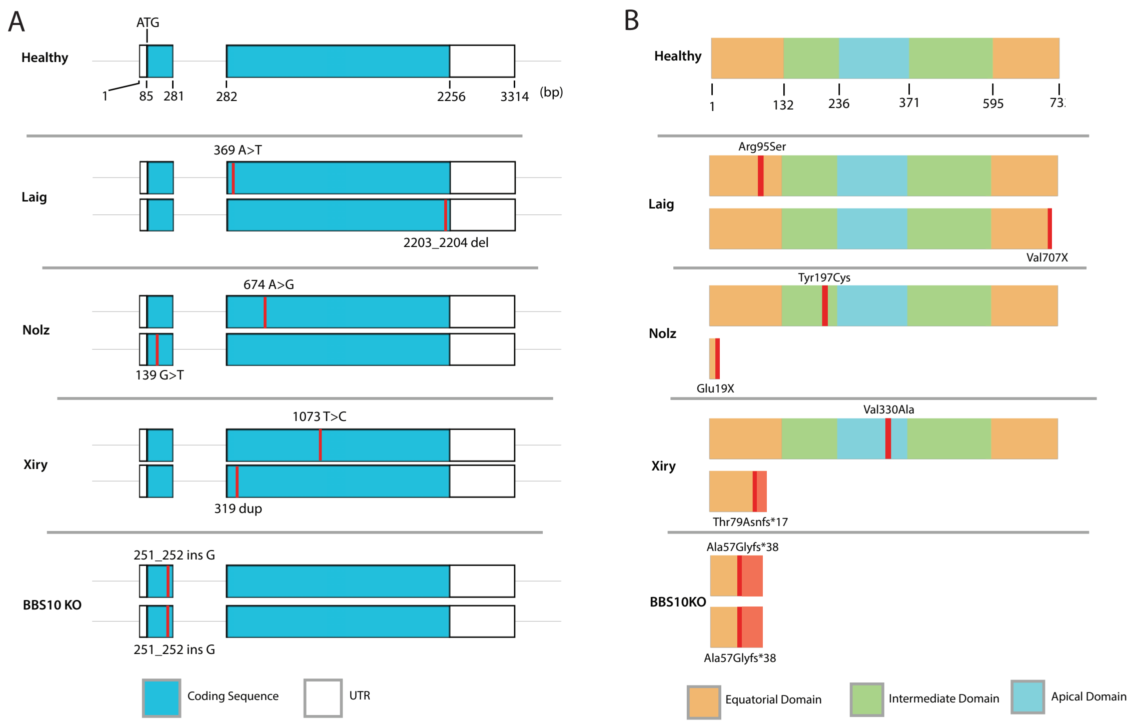
A
ATG
Healthy
1
85
281
282
2256
3314
(bp)
369 A>T
Laig
2203_2204 del
674 A>G
Nolz
139 G>T
1073 T>C
Xiry
319 dup
251_252 ins G
BBS10 KO
251_252 ins G
Coding Sequence
UTR
B
Healthy
1
132
236
371
595
Arg95Ser
Laig
Val707X
Tyr197Cys
Nolz
Glu19X
Val330Ala
Xiry
Thr79Asnfs*17
Ala57Glyfs*38
BBS10KO
Ala57Glyfs*38
Equatorial Domain
Intermediate Domain
Apical Domain
